# The Association Between eHealth Literacy and Health Behaviors During and Since the COVID-19 Pandemic: Systematic Review and Meta-Analysis

**DOI:** 10.2196/94233

**Published:** 2026-07-09

**Authors:** Boyu Ruan, Jiashuai Tian, Xiaoyuan Wang, Dai Su

**Affiliations:** 1 School of Public Health, Capital Medical University, No 10 Xitoutiao Road, You’anmenwai Avenue, Beijing, 100069, China, 8617800852385

**Keywords:** eHealth literacy, health behavior, COVID-19, systematic review, meta-analysis

## Abstract

**Background:**

Since COVID-19, health information seeking, service navigation, and routine care have become increasingly digitally mediated. It remains unclear whether the association between eHealth literacy and health behaviors is consistent across behavioral domains, populations, and analytic frameworks.

**Objective:**

This systematic review and meta-analysis synthesized COVID-19 and post–COVID-19 evidence on the association between eHealth literacy and health behaviors and examined variation across study contexts.

**Methods:**

We conducted a PRISMA (Preferred Reporting Items for Systematic Reviews and Meta-Analyses) 2020-compliant, PROSPERO-registered review (CRD420251009048). PubMed, Embase, Web of Science Core Collection, CINAHL Ultimate, and Scopus were searched from January 1, 2020, through March 27, 2026. Eligible observational studies assessed eHealth literacy and reported analyzable associations with health behavior outcomes collected in 2020 or later. Health behaviors were classified as health decision-making, health-promoting, or health management behaviors. Correlation coefficients, grouped odds ratios (ORs), and continuous ORs were synthesized separately using random-effects models with Knapp-Hartung adjustment. Certainty was assessed using the Grading of Recommendations Assessment, Development, and Evaluation framework.

**Results:**

In total, 19 studies were included: 10 contributed correlation coefficients, 6 grouped ORs, and 3 continuous ORs. In the correlation-based synthesis, higher eHealth literacy was associated with more favorable health behaviors (pooled *r*=0.43, 95% CI 0.36‐0.51; 95% prediction interval 0.22‐0.61), with substantial heterogeneity (*I*^2^=80.50%). In the grouped OR synthesis, higher eHealth literacy was also associated with more favorable health behaviors (pooled OR 2.12, 95% CI 1.47‐3.05; 95% prediction interval 1.11‐4.06), with moderate heterogeneity (*I*^2^=46.33%). In the continuous OR synthesis, all 3 studies showed positive associations, but the pooled effect was not statistically significant (pooled OR 1.07, 95% CI 0.89‐1.30; 95% prediction interval 0.84‐1.37), with very high heterogeneity (*I*^2^=97.98%). Subgroup analyses showed a significant difference only by geriatric status in the grouped OR synthesis. Certainty was low for the grouped OR synthesis and very low for the other 2 syntheses.

**Conclusions:**

Higher eHealth literacy was generally associated with more favorable health behaviors in the correlation-based and grouped OR syntheses, whereas evidence from the continuous OR synthesis was inconclusive. Given the predominantly cross-sectional evidence base, heterogeneity, risk-of-bias concerns, and low to very low certainty, the association should be interpreted as contextual and associative rather than causal or uniform. This review is innovative in synthesizing COVID-19 and post–COVID-19 evidence, applying a functional classification of health behaviors, and analyzing distinct effect measures separately. Unlike previous reviews that summarized the association more broadly, it avoids a single mixed pooled effect and provides a cautious, context-specific interpretation. In practice, interventions should pair eHealth literacy improvement with trustworthy digital services, clinician support, and behavior-specific conditions that help translate digital information into sustained health-related action.

## Introduction

Since COVID-19, digital platforms have become more deeply embedded in health information seeking, service navigation, self-monitoring, and routine care delivery [1-4]. In parallel, the COVID-19 infodemic has increased the volume, speed, and inconsistency of online health information, making the ability to access, evaluate, and apply digital health information more directly relevant to real-world health action [4]. In this context, eHealth literacy should not be treated as a peripheral digital skill, but as a capability with potential behavioral consequences across contemporary health systems.

Norman and Skinner [5] defined eHealth literacy as the ability to seek, find, understand, and appraise health information from electronic sources and apply the knowledge gained to addressing or solving a health problem. This definition remains an appropriate conceptual starting point for examining how digitally mediated information capability may relate to health behavior. However, the measurement literature also shows substantial variation in instrument content, dimensional structure, and construct operationalization across studies, indicating that measurement heterogeneity must be addressed explicitly when synthesizing evidence in this field [6].

In this review, eHealth literacy is conceptualized as a behavior-relevant capability rather than a purely cognitive attribute. The health belief model provides one rationale for this position, as health action is shaped by perceived susceptibility, severity, benefits, barriers, and cues to action [7]. In digital health settings, individuals with higher eHealth literacy may be better able to access relevant information, judge its credibility, and use it to interpret risks and potential benefits, thereby facilitating the translation of information into action.

However, the form of that action should not be assumed to be uniform. The capability, opportunity, motivation—behavior framework conceptualizes behavior as arising from interactions among capability, opportunity, and motivation [8]. In this framework, eHealth literacy is most closely related to capability, but different health behaviors may depend to different degrees on opportunity and motivation as well. Health decision-making behaviors often involve discrete choices that rely more directly on information appraisal, whereas health-promoting and health management behaviors may require sustained action, environmental support, and adaptation to illness-related demands. This informed this review’s classification of outcomes into health decision-making behaviors, health-promoting behaviors, and health management behaviors, and also suggests that the behavioral relevance of eHealth literacy may vary across contexts rather than being determined by capability alone. The key question, therefore, is whether the association between eHealth literacy and health behavior is consistent across these behavioral functions and study contexts.

A central methodological problem in the existing literature is that “health behaviors” are often pooled as a single outcome despite substantial heterogeneity in behavioral function. This is not a trivial classification issue. Discrete preventive decisions, routine lifestyle practices, and sustained condition-related self-management differ in temporality, motivational structure, and informational demands, and they should not automatically be assumed to reflect the same pathway from eHealth literacy to action [8]. For this reason, this review adopts a functional classification of health behaviors, grouping eligible outcomes into health decision-making behaviors, health-promoting behaviors, and health management behaviors. This framework is used to improve conceptual comparability across studies and to support more interpretable subgroup analyses, rather than to imply that all health behaviors operate through a single mechanism.

This review should also be positioned accurately against prior evidence. It is not the first synthesis in this area. Earlier reviews examined health literacy in the eHealth era more broadly [9], summarized the association between eHealth literacy and health-related outcomes among older adults [10], and reviewed determinants and outcomes of eHealth literacy in healthy adults [11]. A recent meta-analysis further reported a positive overall association between eHealth literacy and health-related behaviors [12]. However, further synthesis remains warranted. First, an increasing number of studies conducted during and since the COVID-19 pandemic have emerged in a substantially changed digital health environment, and evidence suggests that digital health literacy is associated with web-based information-seeking patterns and pandemic-related health behaviors in ways that may not be directly comparable with earlier evidence bases [13-15]. Second, although previous reviews considered health-related behaviors, further conceptual clarification is needed to distinguish functional domains of health behavior more clearly. Third, prior quantitative syntheses did not fully address the growing use of multiple effect size types, including grouped odds ratios (ORs), continuous ORs, and correlation coefficients, which are not directly interchangeable and should be synthesized separately. Accordingly, this systematic review and meta-analysis aimed to synthesize evidence on the association between eHealth literacy and health behaviors in studies with data collected during or since the COVID-19 period and to examine this association using a structured health behavior framework and separate syntheses for different effect size types.

## Methods

### Protocol Registration and Reporting Standards

This systematic review and meta-analysis was prospectively registered in PROSPERO (registration CRD420251009048), where the registered protocol is available. The review was conducted and reported in accordance with the PRISMA (Preferred Reporting Items for Systematic Reviews and Meta-Analyses) 2020 statement, and the literature search was reported following the PRISMA-S (Preferred Reporting Items for Systematic Reviews and Meta-Analyses Literature Search Extension) [16,17]. The PRISMA 2020 checklist and PRISMA-S checklist are provided in Checklist 1 and Checklist 2, respectively. The study selection process is presented in the PRISMA 2020 flow diagram. Certainty of evidence for the main quantitative syntheses was assessed using the Grading of Recommendations Assessment, Development, and Evaluation (GRADE) approach at the outcome level [18,19].

### Ethical Considerations

This study was approved by the Institutional Review Board of Capital Medical University (approval 2023SY081). As no individual-level data were collected and all analyses were conducted using publicly available aggregate data, additional informed consent was not required. Detailed information regarding the ethics approval process and supporting documentation is provided in Multimedia Appendix 1.

### Information Sources and Search Strategy

#### Information Sources

We searched PubMed, Embase, Web of Science Core Collection, CINAHL Ultimate, and Scopus from January 1, 2020, to March 27, 2026. No language restrictions were applied at the search stage. Gray literature was not systematically searched or included. To reduce the risk of missing eligible studies, we manually screened the reference lists of all included studies and relevant reviews and conducted forward citation tracking. For feasibility, retrieval was limited to studies published in 2020 or later; however, final eligibility was determined based on the reported timing of data collection rather than publication year alone.

#### Search Strategy

Search strategies combined controlled vocabulary where applicable, including MeSH in PubMed, Emtree in Embase, and subject headings in CINAHL Ultimate, with free-text terms related to eHealth literacy and health behaviors. Database-specific field tags and limits were applied as appropriate. The full search strategies for all databases are provided in Multimedia Appendix 2.

### Eligibility Criteria and Study Selection

#### Eligibility Criteria

We included observational studies in human participants that examined the association between eHealth literacy and health behavior outcomes. To be eligible, a study had to meet all of the following criteria: (1) eHealth literacy was assessed using a named instrument or a clearly described operational measure with an explicit score, (2) the study reported at least 1 health behavior outcome that could be classified into a prespecified behavioral domain, (3) the association between eHealth literacy and the eligible health behavior outcome was reported or could be derived from the available data in a form suitable for quantitative synthesis, and (4) data collection occurred in 2020 or later or the study explicitly stated that data were collected during or since the COVID-19 period.

Eligible study designs included cross-sectional studies. We excluded studies that focused solely on instrument development, adaptation, or validation; did not report an analyzable measure of eHealth literacy; did not include an eligible health behavior outcome; did not assess the association of interest; collected data before 2020; or did not report the timing of data collection. Conference abstracts, meeting abstracts, study protocols, editorials, commentaries, letters, reviews, meta-analyses, scoping reviews, and nonhuman studies were also excluded.

For quantitative synthesis, studies were included if they reported, or allowed derivation of, either ORs or correlation coefficients (*r*). These effect measures were selected because they are the most commonly reported and enable synthesis within comparable data structures. ORs typically correspond to binary outcomes or grouped comparisons, whereas *r* reflects associations between continuous variables. To preserve interpretability and avoid introducing additional assumptions, only studies reporting these effect measures were included in the meta-analysis.

#### Selection Process

All records identified through database searching were imported into EndNote X9 (Clarivate Analytics) for reference management. Duplicate records were removed using the EndNote duplicate-detection function and then checked manually based on title, authors, year, journal, DOI, and abstract before screening. The deduplicated records were then exported for title and abstract screening.

BR and JT independently screened the remaining records by title and abstract against the prespecified eligibility criteria. Records clearly irrelevant at the title or abstract stage were excluded. Reports sought for retrieval were then obtained, and reports not retrieved were recorded. Retrieved reports were assessed for eligibility independently by the same 2 reviewers. Full-text papers excluded at the eligibility stage were recorded with reasons in predefined categories. The study selection process was documented using a PRISMA 2020 flow diagram.

### Outcome Framework, Data Extraction, and Risk of Bias Assessment

#### Data Items

Because the included studies reported heterogeneous behavioral outcomes, eligible outcomes were classified a priori into 3 functional domains: health decision-making behaviors, health-promoting behaviors, and health management behaviors. This classification framework was defined before data extraction and used to improve conceptual comparability across studies.

Health decision-making behaviors were defined as discrete choice-based actions in which individuals use health information to decide whether to initiate a preventive or care-related action. Health-promoting behaviors were defined as routine or repeated actions undertaken to maintain or improve health in the absence of active disease management. Health management behaviors were defined as ongoing self-regulatory actions undertaken to monitor, control, or reduce an existing health condition or risk.

Detailed operational definitions and examples are provided in Table 1. Accordingly, COVID-19 preventive behaviors were classified as health-promoting behaviors when they reflected repeated protective practices, whereas vaccination-related outcomes were classified as health decision-making behaviors because they represented discrete preventive choices.

**Table 1.** Functional classification of health behavior outcomes.

Domain	Operational definition	Core behavioral feature	Typical outcomes
Health decision-making behaviors	Discrete choice-based actions in which individuals use health information to decide whether to initiate a preventive or care-related action	Decision or initiation	Vaccination uptake, screening participation, testing uptake, care-seeking decisions
Health-promoting behaviors	Routine or repeated actions undertaken to maintain or improve health, in the absence of active disease management	General health maintenance or prevention	Physical activity, diet, sleep, smoking cessation, alcohol reduction, stress management
Health management behaviors	Ongoing self-regulatory actions undertaken to monitor or control an existing health condition or risk	Sustained self-regulation	Medication adherence, self-monitoring, symptom tracking, chronic disease management

The following data items were extracted for each eligible study: author and publication year, data collection period, country or region and setting, participant population, study design, eHealth literacy instrument, health behavior scale or measure, specific health behavior outcome, health behavior domain according to the predefined classification framework in Table 1, effect-size framework, effect estimate, 95% CI, sample size, and information required for effect-size transformation and quantitative synthesis. Correlation type was extracted for studies reporting correlation coefficients.

#### Study Risk of Bias Assessment

Methodological quality and risk-of-bias–related concerns were assessed independently by 2 reviewers using a modified Newcastle-Ottawa Scale (NOS) for observational studies [20,21]. The scale included 7 domains: representativeness, sample size justification, nonresponse, exposure assessment, confounding control, outcome assessment, and statistical methods, with a total score ranging from 0 to 10.

Studies were classified as high quality if they scored 8 to 10, moderate quality if they scored 5 to 7, and low quality if they scored 0 to 4. Disagreements were resolved through discussion, with adjudication by a third reviewer when necessary.

### Certainty of Evidence and Statistical Analysis

#### Data Collection Process

A standardized data extraction form was developed and pilot-tested before formal extraction. In total, 2 reviewers independently extracted all data items.

For studies reporting multiple relevant outcomes, a prespecified hierarchical rule was applied to preserve statistical independence. First, the estimate based on the largest analyzable sample was prioritized. Second, if multiple eligible estimates remained, the outcome most closely aligned with the predefined behavioral domains was selected. Third, the estimate judged to be most proximal to the hypothesized pathway linking eHealth literacy to behavior was retained.

If a study reported results for mutually exclusive subgroups derived from distinct participant samples, each subgroup estimate was eligible to enter the synthesis separately. If multiple estimates were derived from overlapping samples, only one estimate was included in the primary analysis to avoid double-counting.

#### Effect Measures

Because the included studies reported different analytic structures, OR-based and correlation-based results were synthesized separately. Within the OR-based synthesis, studies were further classified as grouped OR studies and continuous OR studies according to how eHealth literacy was modeled. Grouped ORs were defined as effect estimates derived from categorical comparisons of eHealth literacy levels, such as low versus high or inadequate versus adequate eHealth literacy. Continuous ORs were defined as effect estimates derived from models in which eHealth literacy was analyzed as a continuous variable, with the OR representing the change in the outcome per unit increase in eHealth literacy. These 2 types of ORs were reported separately because they differ in exposure structure and interpretation. Correlation coefficients were synthesized separately for studies in which both eHealth literacy and health behavior were analyzed on a continuous scale, and the association was reported as a correlation coefficient. These effect measures were not combined because they represent different statistical constructs and are not directly comparable without additional assumptions.

For correlation-based synthesis, correlation coefficients were transformed to Fisher *z* values before pooling and were back-transformed to *r* for presentation [22].

For OR-based synthesis, ORs and their 95% CIs were extracted directly when available. When studies reported effect estimates using the opposite reference category or outcome coding, the estimates were recalculated so that ORs greater than 1 consistently indicated a positive association between higher eHealth literacy and the target health behavior. Corresponding CIs were transformed accordingly.

Detailed transformation procedures are provided in Multimedia Appendix 3.

#### Synthesis Methods

Meta-analyses were conducted in R (version 4.5.2; R Foundation for Statistical Computing). Random-effects models were specified a priori for all quantitative syntheses because clinical and methodological heterogeneity was anticipated across study populations, measurement tools, and behavior outcomes. Between-study variance was estimated using the DerSimonian-Laird method, and Knapp-Hartung adjustment was applied to the CIs and statistical tests of pooled effects [23]. Accordingly, pooled effect tests were based on the 2-tailed *t* distribution rather than conventional Wald-type *z* tests. Heterogeneity was assessed using Cochran *Q*, *I*^2^, and τ^2^. Pooled estimates were reported with 95% CIs, and 95% prediction intervals were calculated where feasible to reflect the expected range of true effects across comparable study settings [24]. For presentation, correlation coefficients were back-transformed from Fisher *z* values to *r*, and ORs were back-transformed from the log(OR) scale to the OR scale.

Subgroup analyses were conducted only when a subgroup included a sufficient number of studies and when the comparison had been prespecified. Additional study-level characteristics extracted for subgroup analyses included country income level, patient status, and geriatric status.

Country income level was classified according to the World Bank income classification [25]. Studies were categorized as being from high-income economies or low- and middle-income economies based on the country in which each study was conducted.

Patient status classified studies as involving either patient populations or general populations. Patient populations included participants with a specific diagnosed health condition, whereas general populations included community-based or nonclinical samples not restricted to a specific disease.

Geriatric status classified studies as involving either older-adult populations or nonolder-adult populations according to the predominant age profile of the study population. Older-adult populations included studies explicitly targeting older adults, studies with a lower age boundary of 65 years or older, and studies described as involving middle-aged and older adults when the original study clearly reflected an older-oriented population. Nonolder-adult populations included younger-adult, middle-aged, and broader mixed-age samples without a primary focus on older adults. Because several included studies did not report subgroup-specific estimates for narrow age bands, this classification was based on the predominant population orientation of the original study rather than on a single rigid age cutoff.

#### Reporting Bias Assessment

Publication bias was evaluated using funnel plots and the Egger regression test when the number of studies was sufficient. When asymmetry was suggested, trim-and-fill analyses were conducted as sensitivity analyses to assess the robustness of pooled estimates.

#### Certainty Assessment

The certainty of evidence was assessed using the GRADE approach for each main quantitative synthesis. Because the included studies reported different effect-size structures, certainty was assessed separately for the correlation-based synthesis, the grouped OR-based synthesis, and the continuous OR-based synthesis. The certainty rating reflected confidence in the estimated association between eHealth literacy and health behavior within each effect-size framework, rather than confidence in a causal effect.

Because the included studies were observational and predominantly cross-sectional, the initial certainty of evidence was rated as low. Certainty was then assessed across the GRADE domains of risk of bias, inconsistency, indirectness, imprecision, and publication bias. Risk of bias judgments considered study-level methodological limitations, including representativeness, nonresponse, exposure and outcome assessment, confounding control, and appropriateness of statistical methods. Inconsistency was judged using the magnitude and direction of effects, *I*^2^, τ^2^, Cochran *Q*, and 95% prediction intervals where available. Indirectness was assessed according to the relevance of the populations, eHealth literacy measures, health behavior outcomes, and study contexts to the review question. Imprecision was assessed using the width of CIs, prediction intervals, the number of contributing studies, and whether the interval estimates included values compatible with no association or materially different interpretations. Publication bias was assessed using funnel plots and Egger regression when the number of studies was sufficient, while also considering the possibility of selective reporting and nonpublication of null findings.

Potential upgrading was considered according to GRADE guidance, including large effect, dose-response gradient, and plausible residual confounding that would reduce the observed association.

### Deviations From the Registered Protocol

Several deviations from the registered protocol should be noted. The most important change was the introduction of a clearer time restriction, such that this review focused on studies with data collected during and since the COVID-19 period. In addition, the framework used to classify health behavior outcomes was refined, different effect-size metrics (grouped ORs, continuous ORs, and correlation coefficients) were synthesized separately, and the search strategy was updated, including changes to information sources, databases, and search terms. As a result of these methodological refinements, some aspects of the final study selection and results differed from those originally described in the registered protocol. These changes were made to improve conceptual clarity, search sensitivity, and the interpretability of the quantitative synthesis.

## Results

### Study Selection

**Figure 1.**
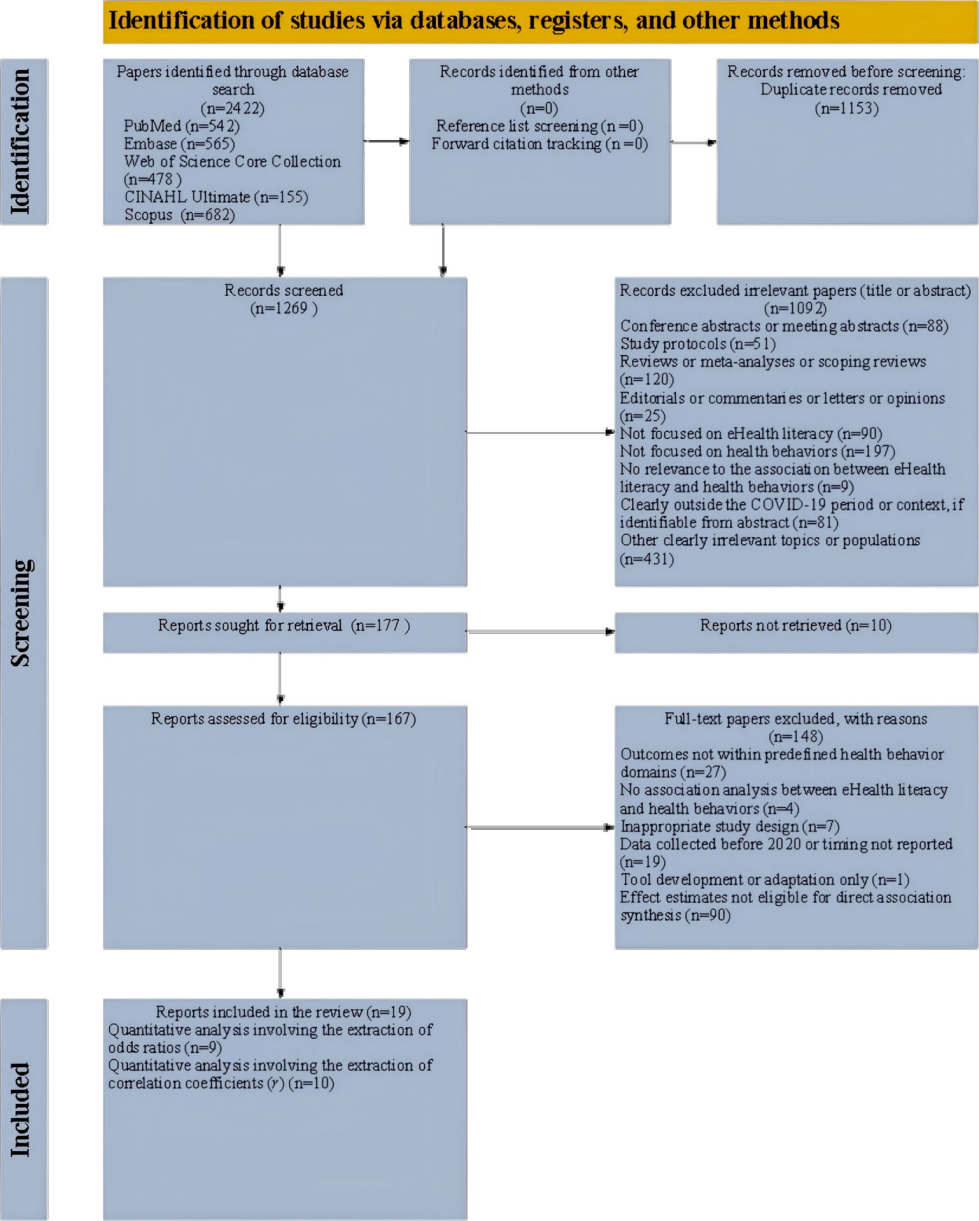
PRISMA flow diagram. PRISMA: Preferred Reporting Items for Systematic Reviews and Meta-Analyses.

The study selection process is illustrated in the PRISMA 2020 flow diagram (Figure 1). A total of 2422 records were identified through database searches, including PubMed (n=542), Embase (n=565), Web of Science Core Collection (n=478), CINAHL Ultimate (n=155), and Scopus (n=682). After removal of 1153 duplicate records, 1269 records remained for title and abstract screening.

During the screening stage, 1092 records were excluded based on title or abstract, including conference abstracts or meeting abstracts (n=88), study protocols (n=51), reviews, meta-analyses, or scoping reviews (n=120), editorials, commentaries, letters, or opinions (n=25), studies not focused on eHealth literacy (n=90), studies not focused on health behaviors (n=197), studies not addressing the association between eHealth literacy and health behaviors (n=9), studies clearly outside the COVID-19 period or context (n=81), and other clearly irrelevant topics or populations (n=431).

A total of 177 reports were sought for full-text retrieval, of which 10 reports were not retrieved. The remaining 167 full-text papers were assessed for eligibility. Of these, 148 papers were excluded for the following reasons: outcomes not within predefined health behavior domains (n=27), no association analysis between eHealth literacy and health behaviors (n=4), inappropriate study design (n=7), data collected before 2020 or timing not reported (n=19), tool development or adaptation only (n=1), and effect estimates not eligible for direct quantitative synthesis (n=90), with detailed classifications provided in Multimedia Appendix 4.

Finally, 19 studies were included in the quantitative synthesis. Among these, 10 studies contributed to the correlation-based quantitative synthesis [14,26-34], and 9 studies contributed to the OR-based quantitative synthesis [35-43].

### Study Characteristics

#### Correlation-Based Synthesis

A total of 10 studies reported correlation coefficients examining the association between eHealth literacy and health behavior outcomes [14,26-34]. These studies were conducted across 4 countries, including China (n=4) [14,26-28], Iran (n=3) [29-31], South Korea (n=2) [32,33], and Turkey (n=1) [34], indicating that the available evidence was concentrated in Asian and Middle Eastern settings. All included studies used a cross-sectional design. Sample sizes ranged from 138 to 1873 participants [14,32]. The majority of studies were conducted in nonpatient populations, including middle school students [32], nursing students [33], medical students [29], university students [14], community residents [30], registered nurses [31], and caregivers of children with type 1 diabetes mellitus [26]. In total, 3 studies focused on patient populations, including individuals with coronary heart disease [28], diabetes [34], and gout [27]. Only 1 study specifically targeted an older population, namely middle-aged and older patients with coronary heart disease aged 45 years and older [28]. eHealth literacy was predominantly assessed using the eHealth Literacy Scale (eHEALS) or its adapted versions. In total, 9 of the 10 studies used eHEALS-based instruments, including Chinese and Persian versions [14,26-32,34], whereas 1 study used a self-designed multidimensional scale incorporating functional, communicative, and critical domains of eHealth literacy [33]. Health behavior outcomes were classified into 2 prespecified functional domains: health-promoting behaviors (n=6) [14,29-33] and health management behaviors (n=4) [26-28,34]. Health-promoting behaviors were assessed using heterogeneous lifestyle-oriented measures, including Health-Promoting Lifestyle Profile–based instruments [29,31,33], the healthy lifestyles tool by Choi et al [32], and COVID-19–related behavioral questionnaires [14,30]. Health management behaviors were primarily assessed in patient populations and included disease-specific self-management and adherence-related behaviors, such as diabetes management [26,34], nonpharmacological adherence in coronary heart disease [28,30], and gout self-management [27]. No study in the correlation-based synthesis contributed to the health decision-making behavior domain. Correlation estimates were reported predominantly as Pearson coefficients (n=8) [14,26,27,29,30,32,34], with 2 studies using Spearman coefficients [28,31].

#### Grouped OR-Based Synthesis

A total of 6 studies reported grouped ORs based on categorical comparisons of eHealth literacy levels in relation to health behavior outcomes [35-40]. These studies were conducted across 5 settings, including China (n=3) [35-37], South Korea (n=1) [38], Saudi Arabia (n=1) [39], and Ethiopia (n=1) [40], indicating that the grouped OR-based evidence was concentrated in Asian settings, with additional representation from the Middle East and Africa. All included studies used a cross-sectional design. Sample sizes ranged from 478 to 6704 participants [35,39]. In total, 3 studies specifically focused on older populations, including older adults with frailty or prefrailty [37], older community populations [35], and general older adults [38]. A total of 2 studies were conducted in patient populations, namely, patients receiving dental care [39] and older participants with frailty or prefrailty [37]. eHealth literacy was measured predominantly using the eHEALS or its adapted Chinese versions. All included studies assessed eHealth literacy using eHEALS or closely related variants, including Chinese-language adaptations [35-40]. In total, 4 studies were classified as health-promoting behaviors [35,36,38,39], 1 study as health management behavior [37], and 1 study as health decision-making behavior [40]. The health-promoting behavior domain included wearing a surgical mask [36], tooth brushing [39], nonsmoking [35], and broader preventive behaviors [38]. The health management behavior domain was represented by medication adherence [37], whereas the health decision-making behavior domain was represented by COVID-19 vaccine uptake [40]. The measurement of health behaviors was heterogeneous. In total, 3 studies did not report a named health behavior scale in the extracted data [36,39,40], whereas the remaining studies used a 4-item Morisky medication adherence scale [37], a mixed measure combining International Physical Activity Questionnaire-Short Form physical activity assessment with self-reported smoking and drinking items [35], and a 10-item COVID-19 preventive behavior scale based on World Health Organization and South Korea Centers for Disease Control and Prevention recommendations [38].

#### Continuous OR-Based Synthesis

A total of 3 studies reported ORs derived from models in which eHealth literacy was analyzed as a continuous variable.

All studies were conducted in Asian settings, including China (n=1) [41] and Vietnam (n=2) [42,43]. All included studies used a cross-sectional design. Sample sizes ranged from 1851 to 5209 participants [41-43], reflecting relatively large study populations.

The study populations were limited to nonpatient groups, including community residents [41], medical staff [42], and nursing undergraduate students [43]. None of the included studies specifically targeted older populations, and all studies were conducted in nonclinical settings. eHealth literacy was assessed consistently across all studies using the eHEALS, including language-adapted versions. Compared with other synthesis groups, the continuous OR-based studies showed minimal variation in exposure measurement.

All included studies were classified under the health-promoting behavior domain. The specific behaviors assessed included physical activity [41], exercise [42], and hand hygiene-related preventive behaviors [43]. Behavioral outcomes were measured using heterogeneous approaches, including self-compiled lifestyle measures [41], classifications of self-reported behavioral changes [42], and single-item frequency-based questions [43]. The characteristics of studies are summarized in Table 2, and full details are provided in Tables S1-S3 in Multimedia Appendix 5 [25-43].

**Table 2.** Characteristics of included studies (n=19).

Author (year)	Study setting	Population	eHEALS^a^	Specific health behavior outcome	NOS^b^ score	Effect size
Yu et al (2023) [26]	China	Primary caregivers of children with type 1 diabetes mellitus	eHEALS, 8 items, Chinese version	Diabetes management behaviors	5	Correlation coefficient
Choi et al (2021) [32]	South Korea	Middle school students	Adapted eHEALS^a^ based on Norman and Skinner [5]	Healthy lifestyle	7	Correlation coefficient
Kwon and Oh (2023) [33]	South Korea	Nursing students	Self-designed scale (31 items, functional, or communicative or critical)	Health-promoting behavior	9	Correlation coefficient
Moradi et al (2025) [31]	Iran	Registered nurses	eHEALS, 8 items	Healthy lifestyle	9	Correlation coefficient
Mousazadeh et al (2025) [29]	Iran	Medical students	eHEALS, 8 items	Healthy lifestyle	8	Correlation coefficient
Rezakhani Moghaddam et al (2022) [30]	Iran	Community residents aged 18 to 65 years	Persian eHEALS, 8 items	Healthy lifestyle	7	Correlation coefficient
Sun et al (2025) [28]	China	Middle-aged and older patients with coronary heart disease aged 45 years and older	eHEALS, 8 items, Chinese version	Nonpharmacological adherence	10	Correlation coefficient
Töyer Şahin and Pehlivan (2026) [34]	Turkey	Patients with diabetes	eHEALS	Disease management	8	Correlation coefficient
Li et al (2021) [14]	China	Chinese university students	Chinese version of eHEALS, 8 items	Healthy lifestyle	7	Correlation coefficient
Qian et al (2024) [27]	China	Male patients with gout	Chinese version of eHEALS, 8 items	Self-management	9	Correlation coefficient
Kalayou and Awol (2022) [40]	Ethiopia	Community adults	eHEALS	Administration of COVID-19 vaccine	7	Grouped OR^c^
Guo et al (2021) [36]	Hong Kong, China	Adults	Chinese eHEALS	Wear a surgical mask	9	Grouped OR
Hakeem et al (2023) [39]	Saudi Arabia	Patients with dental care	eHEALS	Brush	7	Grouped OR
Guo et al (2024) [37]	China	Older participants with frailty or prefrailty	eHEALS Chinese version	Medication adherence	9	Grouped OR
Chau et al (2026) [35]	Hong Kong, China	Older community population	eHEALS	Nonsmoking	8	Grouped OR
Lee et al (2023) [38]	South Korea	General older people	eHEALS	Preventive behavior	7	Grouped OR
Jing et al (2021) [41].	China	Community residents aged 18‐59 years	eHEALS Chinese version	Physical activity	9	Continuous OR
Do et al (2020) [42]	Vietnam	Medical staff	eHEALS	Exercise	9	Continuous OR
Tran et al (2022) [43]	Vietnam	Nursing undergraduate students	eHEALS	Hand hygiene prevention	8	Continuous OR

a eHEALS: eHealth Literacy Scale.

b NOS: Newcastle-Ottawa Scale.

c OR: odds ratio.

### Quality Assessment of Included Studies

**Figure 2.**
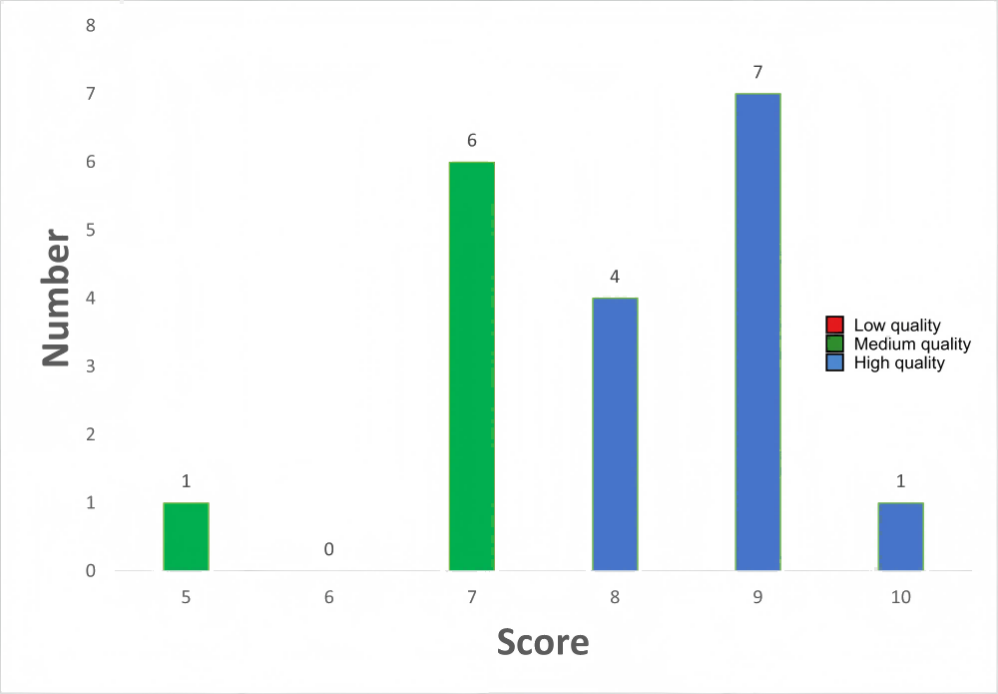
Distribution of modified Newcastle-Ottawa Scale scores among the included studies.

The distribution of modified NOS scores is presented in Figure 2, and detailed scoring results are provided in Tables S1 and S2 in Multimedia Appendix 6 [25-43]. The modified NOS scores ranged from 5 to 10. Among the included studies, 12 were rated as high quality [27,29,31,33,37,41-43], and 7 were rated as moderate quality [14,26,30,32,38-40]. No study was classified as low quality.

Most studies scored well in domains related to exposure and outcome assessment, reflecting the use of named eHealth literacy instruments and clearly defined health behavior measures. Lower scores were more commonly observed in domains related to sample representativeness and control of confounding, as most included studies used cross-sectional designs and convenience-based sampling strategies.

### Certainty of Evidence

Using the GRADE approach, the certainty of evidence for the association between eHealth literacy and health behaviors was rated as low for the grouped OR-based synthesis and very low for the correlation-based and continuous OR-based syntheses. The full GRADE evidence profile and summary of findings table are provided in Multimedia Appendix 7.

Because all included studies were observational and predominantly cross-sectional, the initial certainty of evidence was low. For the grouped OR-based synthesis, the certainty was not rated down further because heterogeneity was moderate, the CI and prediction interval remained consistent with a positive association, and the additional concerns identified were not judged serious enough to warrant further downgrading beyond the initial low rating for observational evidence.

In contrast, the certainty of evidence for the correlation-based synthesis was rated down to very low because of substantial inconsistency across studies. For the continuous OR-based synthesis, the certainty was also rated down to very low because of very high heterogeneity and serious imprecision, mainly related to the small number of contributing studies and interval estimates compatible with no association. No synthesis was upgraded because the criteria for upgrading were not met, including a convincing large effect, a clear dose-response gradient, or plausible residual confounding that would reduce the observed association.

### Results of Syntheses

Because included studies reported associations using different effect size metrics (correlation coefficients, grouped ORs, and continuous ORs), separate meta-analyses were conducted for each effect size framework to avoid inappropriate pooling of heterogeneous measures.

#### Correlation-Based Synthesis

**Figure 3.**
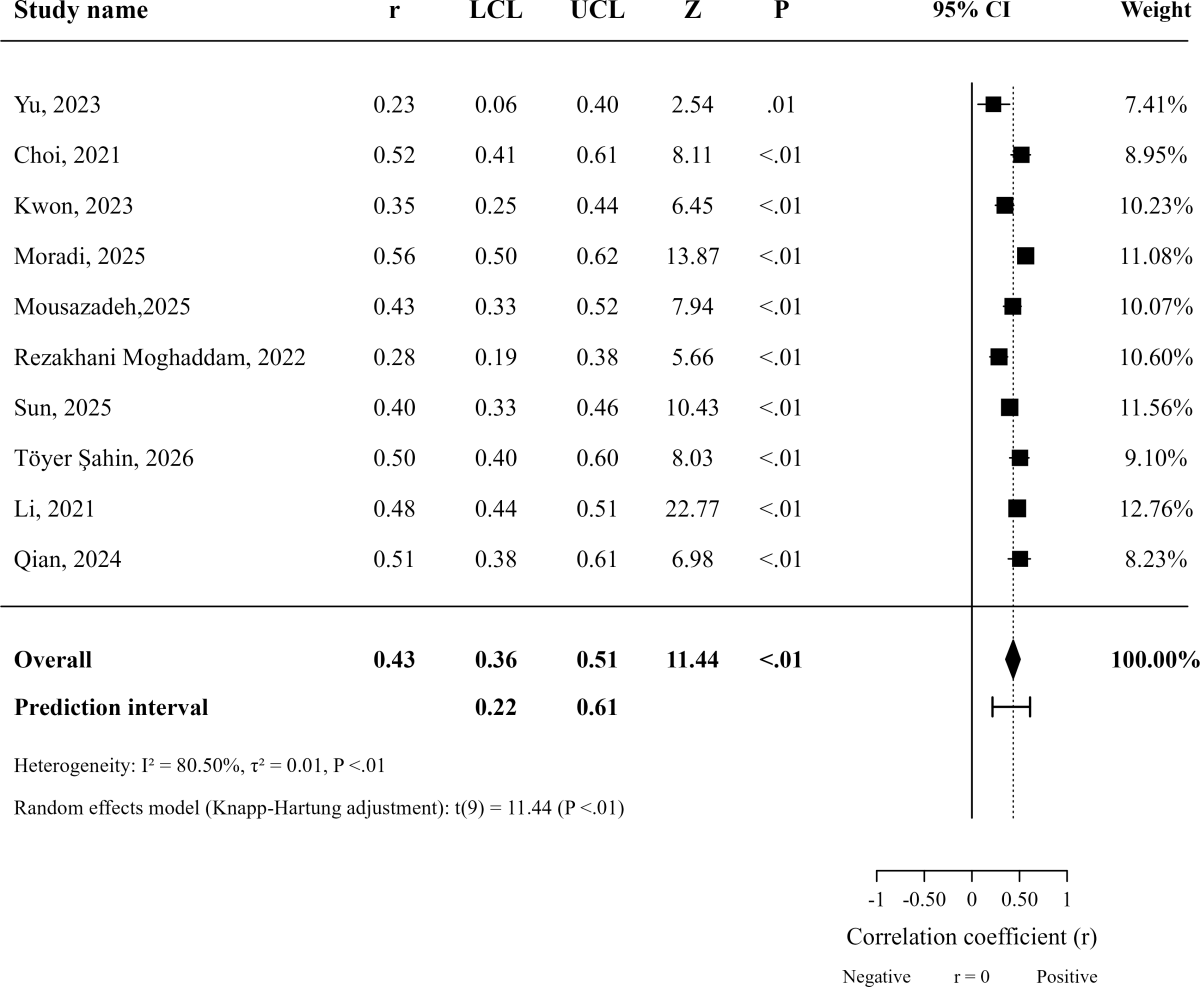
Forest plot of correlation coefficients for the association between eHealth literacy and health behaviors. Squares indicate study-specific correlation coefficients, with size proportional to random-effects weight; horizontal lines indicate 95% CIs; the diamond indicates the pooled estimate from the random-effects model with Knapp-Hartung adjustment; and the thick horizontal line below the diamond indicates the 95% prediction interval. LCL and UCL indicate the lower and upper confidence limits, respectively. Values to the right of 0 indicate that higher eHealth literacy is associated with more favorable health behavior [14,26-34].

In total, 10 studies contributed correlation coefficients. Using a random-effects model with Knapp-Hartung adjustment, the pooled estimate showed a moderate positive average association between eHealth literacy and health-related behaviors (*r*=0.43, 95% CI 0.36‐0.51; *t*_9_=11.44; *P*<.001; Figure 3). The 95% CI indicates that the average pooled association was positive and statistically significant. Between-study heterogeneity was substantial (*I*^2^=80.50%; τ^2^=0.01; *P*<.001), indicating considerable variability in the magnitude of the association across studies. The 95% prediction interval ranged from 0.22 to 0.61, suggesting that the true association in future comparable settings would also be expected to remain positive, although its magnitude may vary meaningfully across contexts. Therefore, the pooled estimate should be interpreted as an average association across heterogeneous study contexts rather than as evidence of a single common effect.

#### Grouped OR-Based Synthesis

**Figure 4.**
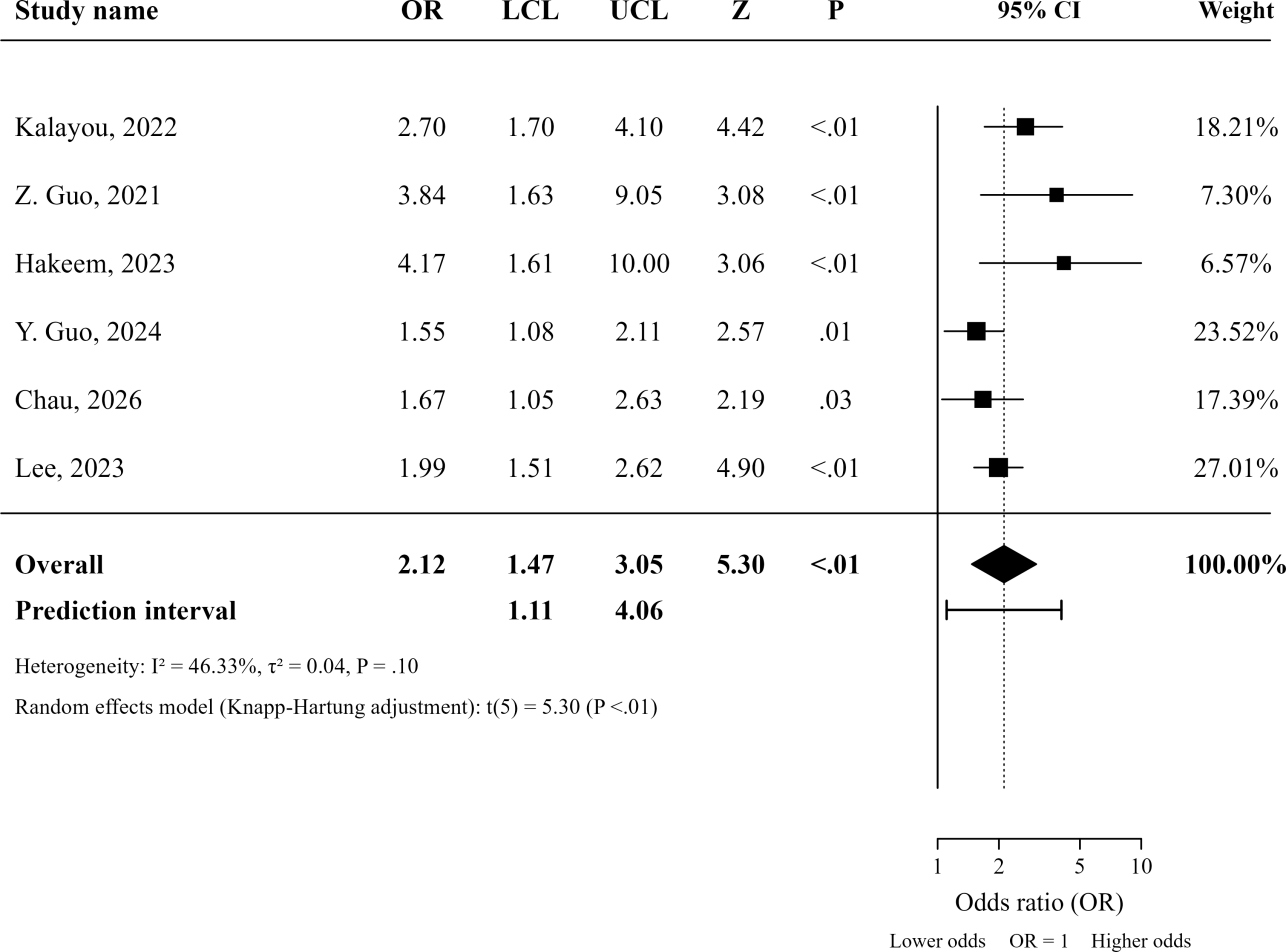
Forest plot of grouped odds ratios (ORs) for the association between eHealth literacy and health behaviors. Squares indicate study-specific grouped ORs, with size proportional to random-effects weight; horizontal lines indicate 95% CIs; the diamond indicates the pooled estimate from the random-effects model with Knapp-Hartung adjustment; and the thick horizontal line below the diamond indicates the 95% prediction interval. LCL and UCL indicate the lower and upper confidence limits, respectively. ORs greater than 1.00 indicate higher odds of favorable health behavior among participants with higher versus lower eHealth literacy [35-40].

In total, 6 studies reported grouped ORs. Using a random-effects model with Knapp-Hartung adjustment, the pooled estimate showed a positive average association between higher eHealth literacy and favorable health-related behaviors (OR 2.12, 95% CI 1.47‐3.05; *t*_5_=5.30; *P*<.001; Figure 4). The 95% CI indicates that the average pooled effect was statistically significant. Heterogeneity was moderate (*I*^2^=46.33%; τ^2^=0.04; *P*=.10), suggesting some between-study variability but less inconsistency than in the correlation-based synthesis. The 95% prediction interval ranged from 1.11 to 4.06 and remained above the null value of 1.00, suggesting that the true effect in comparable settings was compatible with a positive association. However, this should be interpreted cautiously because only 6 studies contributed to this synthesis.

#### Continuous OR-Based Synthesis

**Figure 5.**
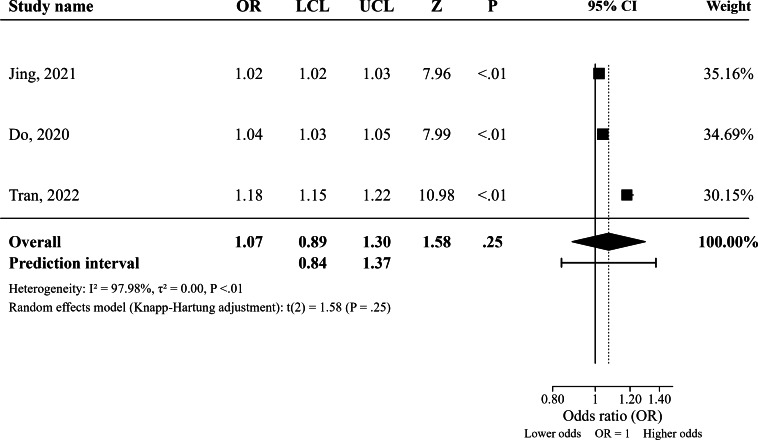
Forest plot of continuous odds ratios (ORs) for the association between eHealth literacy and health behaviors. Squares indicate study-specific continuous ORs, with size proportional to random-effects weight; horizontal lines indicate 95% CIs; the diamond indicates the pooled estimate from the random-effects model with Knapp-Hartung adjustment; and the thick horizontal line below the diamond indicates the 95% prediction interval. LCL and UCL indicate the lower and upper confidence limits, respectively. ORs greater than 1.00 indicate higher odds of favorable health behavior per unit increase in eHealth literacy [41-43].

In total, 3 studies reported continuous ORs. All 3 individual studies showed positive associations, with study-specific 95% CIs above the null value of 1.00. However, when synthesized using a random-effects model with Knapp-Hartung adjustment, the pooled average effect was not statistically significant (OR 1.07, 95% CI 0.89‐1.30; *t*_2_=1.58; *P*=.25; Figure 5). The 95% CI crossed the null value, indicating uncertainty around the average pooled effect after accounting for the small number of studies and between-study variability. Heterogeneity was very high (*I*^2^=97.98%; τ^2^<0.01; *P*<.001), suggesting that the magnitude of association varied markedly across studies despite the consistent positive direction of individual estimates. The 95% prediction interval ranged from 0.84 to 1.37 and also crossed the null value of 1.00, indicating that the true effect in future comparable settings could plausibly range from little or no association to a positive association. Therefore, this synthesis should be interpreted as showing a consistent positive direction at the study level but insufficient certainty regarding the average pooled effect. Given the limited number of studies and the very high heterogeneity, subgroup analysis was not conducted for this synthesis.

### Reporting Bias Assessment

Reporting biases were assessed using funnel plots and Egger regression where appropriate. Visual inspection of funnel plots was performed for the 3 syntheses (Figures S1-S3 in Multimedia Appendix 8). The funnel plot for the correlation-based synthesis showed slight visual asymmetry. However, interpretation of the funnel plots for the grouped OR and continuous OR syntheses was limited because only 6 and 3 studies, respectively, were included.

Egger regression was conducted for the correlation-based synthesis, which included 10 studies. The test did not show statistically significant evidence of small-study effects (intercept=−1.37; SE 1.76; *t*_8_=−0.78; *P*=.46). Because only 6 studies were available for the grouped OR synthesis, Egger regression was performed only as an exploratory analysis. This exploratory test also did not show statistically significant evidence of small-study effects (intercept=2.34; SE 1.16; *t*_4_=2.03; *P*=.11). No formal funnel plot asymmetry test was interpreted for the continuous OR synthesis because only 3 studies were available, making such tests statistically underpowered and unreliable.

Overall, no clear statistical evidence of small-study effects was detected in the syntheses for which Egger regression was applied. Nevertheless, because the number of studies was limited, particularly for the grouped OR and continuous OR syntheses, reporting biases could not be ruled out.

### Subgroup Analysis

Subgroup analyses were performed for the grouped OR-based synthesis and the correlation-based synthesis by income level, patient status, geriatric status, and health behavior domain. Subgroup analyses were not conducted for the continuous OR-based synthesis because only 3 studies were available, all of which contributed to the health-promoting behavior domain, which was considered insufficient for meaningful subgroup comparison. Detailed statistical parameters for subgroup analyses in the correlation-based and grouped OR-based syntheses, as well as the overall meta-analytic parameters for the continuous OR-based synthesis, are provided in Multimedia Appendix 9.

In the correlation-based synthesis, positive correlations were observed across all examined subgroups. For geriatric status, the pooled correlation coefficient was 0.40 (95% CI 0.33‐0.46) in older-adult populations and 0.44 (95% CI 0.37‐0.50) in younger or mixed-age populations. For patient status, the pooled *r* was 0.46 (95% CI 0.37‐0.53) in patient populations and 0.42 (95% CI 0.34‐0.50) in general populations. For income level, the pooled *r* was 0.46 (95% CI 0.39‐0.53) in high-income economies and 0.41 (95% CI 0.31‐0.51) in low- and middle-income economies. For the health behavior domain, the pooled *r* was 0.44 (95% CI 0.36‐0.52) for health-promoting behaviors and 0.42 (95% CI 0.31‐0.52) for health management behaviors. No correlation-based study contributed to the health decision-making behavior domain (Table 3; Table S1 in Multimedia Appendix 9). Formal tests for between-subgroup differences were not statistically significant for income level, patient status, geriatric status, or health behavior domain in the correlation-based synthesis, suggesting that these study-level characteristics did not explain a substantial proportion of the between-study heterogeneity.

**Table 3.** Subgroup-specific pooled associations and tests for between-subgroup differences.

Characteristic and subgroup	Grouped OR^a^ studies, n	Pooled OR (95% CI)	QM (OR)	*P* value (OR)	Correlation studies, n	Pooled *r* (95% CI)	QM (*r*)	*P* value (*r*)
Income level								
High-income economies	4	2.25 (1.58‐3.20)	0.28	.60	4	0.46 (0.39‐0.53)	2.97	.08
Low- and middle-income economies	2	2.01 (1.17‐3.45)	—^b^	—	6	0.41 (0.31‐0.51)	—	—
Patient status								
Patient populations	2	2.31 (0.89‐5.99)	1.09	.30	3	0.46 (0.37‐0.53)	0.19	.67
General populations	4	2.18 (1.68‐2.83)	—	—	7	0.42 (0.34‐0.50)	—	—
Geriatric status								
Older-adult populations	2	1.59 (1.21‐2.08)	4.7	.03	1	0.40 (0.33‐0.46)	2.82	.09
Nonolder-adult populations	4	2.55 (1.85‐3.51)	—	—	9	0.44 (0.37‐0.50)	—	—
Health behavior domain								
Health decision-making behaviors	1	2.70 (1.74‐4.19)	4.14	.13	0	—	2.19	.14
Health-promoting behaviors	4	2.25 (1.58‐3.20)	—	—	6	0.44 (0.36‐0.52)	—	—
Health management behaviors	1	1.55 (1.11‐2.17)	—	—	4	0.42 (0.31‐0.52)	—	—

a OR: odds ratio.

b Not available.

In the grouped OR-based synthesis, positive associations between higher eHealth literacy and health behaviors were also observed across most subgroups. For geriatric status, the pooled OR was 1.59 (95% CI 1.21‐2.08) in older-adult populations and 2.55 (95% CI 1.85‐3.51) in younger or mixed-age populations. For patient status, the pooled OR was 2.31 (95% CI 0.89‐5.99) in patient populations and 2.18 (95% CI 1.68‐2.83) in general populations. For income level, the pooled OR was 2.25 (95% CI 1.58‐3.20) in high-income economies and 2.01 (95% CI 1.17‐3.45) in low- and middle-income economies. For the health behavior domain, the pooled OR was 2.70 (95% CI 1.74‐4.19) for health decision-making behaviors, 2.25 (95% CI 1.58‐3.20) for health-promoting behaviors, and 1.55 (95% CI 1.11‐2.17) for health management behaviors (Table 3; Table S2 in Multimedia Appendix 9). Formal tests for between-subgroup differences showed a statistically significant difference only for geriatric status (QM=4.70; *P*=.03), whereas income level, patient status, and health behavior domain were not significant moderators. This pattern suggests that geriatric status may be a potential source of heterogeneity, but this finding should be considered exploratory because it was based on a small number of studies and study-level classifications. The overall meta-analytic parameters for the continuous OR-based synthesis are reported separately in Table S3 in Multimedia Appendix 9.

Across subgroup strata, the overall direction of association remained positive, but several subgroup estimates were based on a small number of studies and should therefore be interpreted cautiously. In addition, although measurement heterogeneity was considered, its likely impact was limited because the vast majority of included studies assessed eHealth literacy using eHEALS, with only 1 study using a different instrument. Therefore, differences in eHealth literacy measurement were unlikely to be a major driver of the observed heterogeneity, which more plausibly reflects variation in study populations, settings, and behavioral outcomes. Accordingly, the pooled estimates, particularly those from the correlation-based and continuous OR-based syntheses, should be interpreted as average effects across heterogeneous study contexts rather than as single common effects applicable to all settings.

## Discussion

### Interpretation of Findings

This systematic review and meta-analysis examined whether eHealth literacy was associated with health behaviors in studies conducted during and since the COVID-19 period and whether this association varied across behavioral domains and analytic frameworks. The findings were interpreted according to 3 considerations: heterogeneity, risk of bias, and certainty of evidence [18,19,21].

Overall, higher eHealth literacy was generally associated with more favorable health behaviors in the correlation-based and grouped OR-based syntheses, whereas evidence from the continuous OR-based synthesis was less certain after Knapp-Hartung adjustment. In the correlation-based synthesis, the pooled average association was moderate and positive, but heterogeneity was substantial. In the grouped OR-based synthesis, the association was also positive and showed moderate heterogeneity, providing the most consistent quantitative evidence among the 3 effect-size frameworks. In contrast, although all 3 continuous OR studies reported positive study-level associations, the pooled average effect was not statistically significant after Knapp-Hartung adjustment and heterogeneity was very high. These heterogeneity patterns indicate that the pooled estimates should be interpreted as average associations across heterogeneous study contexts, rather than as evidence of a single stable relationship between eHealth literacy and health behaviors. This interpretation is consistent with conceptual work, emphasizing that digital and eHealth literacy are context-dependent constructs shaped by informational, social, and service environments [44,45].

In subgroup analyses, geriatric status showed a statistically significant subgroup difference in the grouped OR-based synthesis, whereas country income level, patient status, and health behavior domain were not statistically significant moderators. This suggests that age structure may partly explain differences in the association, but this finding should be interpreted cautiously because subgroup analyses were based on a limited number of studies and study-level classifications. Although no included study was rated as low quality, several NOS domains indicated remaining methodological limitations, particularly in sample representativeness, nonresponse, and confounding control. These limitations may have introduced selection-related bias and residual confounding in some studies. They are especially relevant because most included studies were cross-sectional, a design that limits temporal ordering and causal inference [46,47]. In accordance with GRADE guidance, the certainty of evidence was low for the grouped OR-based synthesis and very low for the correlation-based and continuous OR-based syntheses. The grouped OR-based synthesis was not downgraded beyond the initial low certainty assigned to observational evidence. The correlation-based synthesis was downgraded because of substantial inconsistency, whereas the continuous OR-based synthesis was downgraded because of very high heterogeneity and serious imprecision. Accordingly, the findings should be interpreted as suggestive evidence of a generally positive but context-dependent association, rather than as definitive evidence of a uniform or causal effect.

### Limitations

First, all included studies were observational and predominantly cross-sectional. Because cross-sectional studies measure exposure and outcome at the same time, they cannot establish temporal ordering or causal direction [46]. Therefore, these findings cannot determine whether higher eHealth literacy leads to healthier behaviors, whether healthier individuals develop higher eHealth literacy, or whether both reflect shared determinants. This interpretation is consistent with STROBE (Strengthening the Reporting of Observational Studies in Epidemiology) guidance on transparent reporting of observational designs rather than causal claims [47].

Second, heterogeneity remained substantial in the correlation-based synthesis and very high in the continuous OR-based synthesis. This heterogeneity was not fully explained by prespecified subgroup variables and likely reflects variation in outcome definitions, health behavior measurement approaches, digital health contexts, and unmeasured study-level factors. Accordingly, the pooled estimates should be interpreted as average associations across heterogeneous study contexts rather than as stable effects applicable to all populations or behavioral domains.

Third, although no study was rated as low quality, several modified NOS domains indicated remaining methodological limitations, particularly in sample representativeness, nonresponse, and confounding control, as shown in Multimedia Appendix 6. These limitations may have introduced selection-related bias and residual confounding in some studies.

Fourth, the certainty of evidence was limited. According to the GRADE assessment, certainty was low for the grouped OR-based synthesis and very low for the correlation-based and continuous OR-based syntheses [18,19]. The grouped OR-based synthesis was not downgraded beyond the initial low certainty assigned to observational evidence. The correlation-based synthesis was downgraded mainly because of substantial inconsistency, whereas the continuous OR-based synthesis was downgraded because of very high heterogeneity and serious imprecision related to the small number of studies and interval estimates compatible with no association. Therefore, the findings should be interpreted as suggestive and hypothesis-generating rather than as definitive evidence of a causal relationship.

Finally, the quantitative synthesis was limited to studies that reported extractable correlation coefficients or ORs, as meta-analysis requires effect estimates that can be computed on a common and interpretable scale [48,49]. Studies examining relevant associations but not reporting effect estimates suitable for direct synthesis were therefore excluded from the meta-analysis, which may have affected the comprehensiveness of the quantitative evidence [48,49]. In addition, although most studies used eHEALS or closely related instruments to assess eHealth literacy [5,6], health behavior outcomes were measured heterogeneously, suggesting that outcome-side variation may have contributed substantially to between-study heterogeneity [22,24].

### Implications for Practice and Research

Taken together, these findings suggest that eHealth literacy may be an important supportive capability for health-related action in contemporary digital health settings, but it should not be interpreted as a sufficient stand-alone determinant of behavior change. This interpretation is consistent with behavior change theory, which emphasizes that capability must interact with opportunity and motivation to produce behavior change [8]. In practice, the behavioral relevance of eHealth literacy is likely to depend on whether digital health information and services are accessible, understandable, usable, and connected to practical opportunities for action [50-53]. It is also shaped by broader digital health inequalities, implementation barriers, patient portal contexts, access to electronic health records, and continuity of digitally supported care [54-57]. Therefore, interventions aiming to improve health behaviors should not rely solely on improving eHealth literacy. Instead, they should combine eHealth literacy enhancement with behavior-specific strategies, accessible and user-centered digital services, clinician support, continuity of care, and structural resources that help individuals translate digital information into sustained health-related action [52-57].

Future research should use more robust study designs to clarify the temporal ordering and potential causal pathways between eHealth literacy and health behaviors. Longitudinal and intervention studies are particularly needed to determine whether improvements in eHealth literacy lead to subsequent and sustained changes in behavior. Researchers should report sufficient data for effect-size synthesis, including adjusted and unadjusted estimates where possible, and should clearly specify whether eHealth literacy is modeled as a continuous or categorical exposure. Future studies should also distinguish health behavior domains more explicitly, because eHealth literacy may operate differently across decision-making, health-promoting, and health management behaviors [52,53]. More evidence is needed from diverse geographic regions, patient and nonpatient populations, older and nonolder adults, and underrepresented behavioral domains, given persistent digital health inequalities and implementation challenges across settings [54-56]. Larger numbers of studies within each subgroup would allow more reliable examination of whether age structure, behavior type, patient status, country income level, or measurement approach explains heterogeneity.

### Conclusions

Higher eHealth literacy was generally associated with more favorable health behaviors in the correlation-based and grouped OR-based syntheses, whereas evidence from the continuous OR-based synthesis was inconclusive after Knapp-Hartung adjustment. Because the evidence base was predominantly cross-sectional, heterogeneous, and limited by risk-of-bias concerns, and because the certainty of evidence was low to very low, this association should be interpreted as suggestive, contextual, and noncausal rather than uniform or definitive.

The innovation of this review lies in synthesizing evidence from the COVID-19 and post–COVID-19 period, applying a functional classification of health behaviors, and separating correlation coefficients, grouped ORs, and continuous ORs rather than combining statistically distinct effect measures into a single pooled estimate. This distinguishes this review from earlier syntheses that examined eHealth literacy and health-related behaviors more broadly but did not provide the same separation by behavioral function and effect-size framework. By combining functional behavioral classification, separate quantitative syntheses, prediction intervals, and GRADE assessment, this review provides a more cautious and context-specific interpretation of the evidence: eHealth literacy is generally associated with more favorable health behaviors in the correlation-based and grouped OR-based syntheses, but the strength and certainty of this association depend on the effect-size framework, behavioral outcome, and study population.

The practical implication is that eHealth literacy should be treated as an important supportive capability, not as a stand-alone solution for behavior change. Interventions aiming to improve health behaviors should not only raise eHealth literacy but also ensure that digital health information and services are trustworthy, usable, accessible, and connected to clinical and social support. Clinician guidance, continuity of care, behavior-specific tools, and feasible opportunities for action are needed to help individuals translate digital information into sustained health-related behavior. Future longitudinal and intervention studies should clarify temporal ordering, test causal pathways, and determine which combinations of eHealth literacy support and service-level conditions produce durable improvements across decision-making, health-promoting, and health management behaviors.

## Supplementary material

10.2196/94233 Multimedia Appendix 1 PRISMA-compliant ethics approval statement.

10.2196/94233 Multimedia Appendix 2 Search strategy.

10.2196/94233 Multimedia Appendix 3 Conversion of reported statistics.

10.2196/94233 Multimedia Appendix 4 Studies reporting effect estimates not eligible for direct association synthesis.

10.2196/94233 Multimedia Appendix 5 Included effect characteristics.

10.2196/94233 Multimedia Appendix 6 Table of Newcastle-Ottawa Scale score.

10.2196/94233 Multimedia Appendix 7 Grading of Recommendations Assessment, Development, and Evaluation summary of findings for the association between eHealth literacy and health behaviors.

10.2196/94233 Multimedia Appendix 8 Funnel plots for assessment of small-study effects across different effect measures.

10.2196/94233 Multimedia Appendix 9 Subgroup statistics.

10.2196/94233 Checklist 1 PRISMA checklist.

10.2196/94233 Checklist 2 PRISMA-S extension checklist.

## Abbreviations

eHEALS: eHealth Literacy Scale
GRADE: Grading of Recommendations Assessment, Development, and Evaluation
NOS: Newcastle-Ottawa Scale
OR: odds ratio
PRISMA: Preferred Reporting Items for Systematic Reviews and Meta-Analyses
PRISMA-S: Preferred Reporting Items for Systematic Reviews and Meta-Analyses Literature Search Extension
STROBE: Strengthening the Reporting of Observational Studies in Epidemiology
