# Supplementary material for: The Association Between eHealth Literacy and Health Behaviors During and Since the COVID-19 Pandemic: Systematic Review and Meta-Analysis
Source: J Med Internet Res. 2026 Jul 9;28:e94233. doi: 10.2196/94233 (PMC13348804; doi:10.2196/94233)
Supplement: Multimedia Appendix 1 [file jmir-v28-e94233-s001.docx]

Ethical Approval:
This study was conducted as a secondary analysis of existing published literature and did not involve direct human subject participation or primary data collection. The protocol was reviewed and registered under the ethical approval granted by the Medical Ethics Committee of Capital Medical University (Approval No. 2023SY081, dated March 1, 2023). The committee waived the requirement for additional ethical review, as the study exclusively utilized anonymized and publicly available data.

PRISMA Compliance:
This review adheres to the PRISMA (Preferred Reporting Items for Systematic Reviews and Meta-Analyses) guidelines. The study protocol was registered in [PROSPERO/Open Science Framework] (As of now, our study submitted to the PROSPERO registration platform (ID: 1009048) is still under review and remains in the registration queue. The registration was initially submitted on March 21, 2025.).
