# Supplementary material for: The Association Between eHealth Literacy and Health Behaviors During and Since the COVID-19 Pandemic: Systematic Review and Meta-Analysis
Source: J Med Internet Res. 2026 Jul 9;28:e94233. doi: 10.2196/94233 (PMC13348804; doi:10.2196/94233)
Supplement: Multimedia Appendix 2 [file jmir-v28-e94233-s002.docx]

**1. PubMed**

(

"eHealth Literacy"[tiab]

OR "electronic health literacy"[tiab]

OR "digital health literacy"[tiab]

OR "online health literacy"[tiab]

OR "electronic health information literacy"[tiab]

OR "internet health literacy"[tiab]

OR "web-based health literacy"[tiab]

OR "web based health literacy"[tiab]

OR "mobile health literacy"[tiab]

OR "mHealth literacy"[tiab]

OR "digital health competence"[tiab]

OR "digital health competencies"[tiab]

OR eHEALS[tiab]

OR eHLQ[tiab]

OR DHLI[tiab]

)

AND

(

"Health Behavior"[Mesh]

OR "Self Care"[Mesh]

OR "Medication Adherence"[Mesh]

OR "Help-Seeking Behavior"[Mesh]

OR "health behavior*"[tiab]

OR "health behaviour*"[tiab]

OR "health-promoting behavior*"[tiab]

OR "health promoting behavior*"[tiab]

OR "health promotion behavior*"[tiab]

OR "health-related behavior*"[tiab]

OR "health related behavior*"[tiab]

OR "lifestyle behavior*"[tiab]

OR "lifestyle behaviour*"[tiab]

OR "healthy lifestyle"[tiab]

OR "health management"[tiab]

OR "self-management"[tiab]

OR "self management"[tiab]

OR "self-care"[tiab]

OR "self care"[tiab]

OR "medication adherence"[tiab]

OR "treatment adherence"[tiab]

OR "health information seeking"[tiab]

OR "help-seeking"[tiab]

OR "help seeking"[tiab]

OR "preventive behavior*"[tiab]

OR "preventive behaviour*"[tiab]

OR "vaccine uptake"[tiab]

OR "screening participation"[tiab]

OR "physical activity"[tiab]

OR exercise[tiab]

OR "physical exercise"[tiab]

)

AND

("2020/01/01"[Date - Publication] : "2026/03/27"[Date - Publication])

**2. Embase**

(

'ehealth literacy':ti,ab,kw

OR 'electronic health literacy':ti,ab,kw

OR 'digital health literacy':ti,ab,kw

OR 'online health literacy':ti,ab,kw

OR 'electronic health information literacy':ti,ab,kw

OR 'internet health literacy':ti,ab,kw

OR 'web-based health literacy':ti,ab,kw

OR 'web based health literacy':ti,ab,kw

OR 'mobile health literacy':ti,ab,kw

OR 'mhealth literacy':ti,ab,kw

OR 'digital health competence':ti,ab,kw

OR 'digital health competencies':ti,ab,kw

OR eHEALS:ti,ab,kw

OR eHLQ:ti,ab,kw

OR DHLI:ti,ab,kw

)

AND

(

'health behavior'/exp

OR 'self care'/exp

OR 'medication compliance'/exp

OR 'help seeking behavior'/exp

OR 'health behavior*':ti,ab,kw

OR 'health behaviour*':ti,ab,kw

OR 'health-promoting behavior*':ti,ab,kw

OR 'health promoting behavior*':ti,ab,kw

OR 'health promotion behavior*':ti,ab,kw

OR 'health-related behavior*':ti,ab,kw

OR 'health related behavior*':ti,ab,kw

OR 'lifestyle behavior*':ti,ab,kw

OR 'lifestyle behaviour*':ti,ab,kw

OR 'healthy lifestyle':ti,ab,kw

OR 'health management':ti,ab,kw

OR 'self-management':ti,ab,kw

OR 'self management':ti,ab,kw

OR 'self-care':ti,ab,kw

OR 'self care':ti,ab,kw

OR 'medication adherence':ti,ab,kw

OR 'treatment adherence':ti,ab,kw

OR 'health information seeking':ti,ab,kw

OR 'help-seeking':ti,ab,kw

OR 'help seeking':ti,ab,kw

OR 'preventive behavior*':ti,ab,kw

OR 'preventive behaviour*':ti,ab,kw

OR 'vaccine uptake':ti,ab,kw

OR 'screening participation':ti,ab,kw

OR 'physical activity':ti,ab,kw

OR exercise:ti,ab,kw

OR 'physical exercise':ti,ab,kw

)

AND [2020-2026]/py

**3. CINAHL Ultimate**

(

(

TI "eHealth literacy" OR AB "eHealth literacy"

OR TI "electronic health literacy" OR AB "electronic health literacy"

OR TI "digital health literacy" OR AB "digital health literacy"

OR TI "online health literacy" OR AB "online health literacy"

OR TI "electronic health information literacy" OR AB "electronic health information literacy"

OR TI "internet health literacy" OR AB "internet health literacy"

OR TI "web-based health literacy" OR AB "web-based health literacy"

OR TI "web based health literacy" OR AB "web based health literacy"

OR TI "mobile health literacy" OR AB "mobile health literacy"

OR TI "mHealth literacy" OR AB "mHealth literacy"

OR TI "digital health competence" OR AB "digital health competence"

OR TI "digital health competencies" OR AB "digital health competencies"

OR TI eHEALS OR AB eHEALS

OR TI eHLQ OR AB eHLQ

OR TI DHLI OR AB DHLI

)

AND

(

MH "Health Behavior+"

OR MH "Self Care+"

OR MH "Medication Adherence"

OR TI "health behavior*" OR AB "health behavior*"

OR TI "health behaviour*" OR AB "health behaviour*"

OR TI "health-promoting behavior*" OR AB "health-promoting behavior*"

OR TI "health promoting behavior*" OR AB "health promoting behavior*"

OR TI "health promotion behavior*" OR AB "health promotion behavior*"

OR TI "health-related behavior*" OR AB "health-related behavior*"

OR TI "health related behavior*" OR AB "health related behavior*"

OR TI "lifestyle behavior*" OR AB "lifestyle behavior*"

OR TI "lifestyle behaviour*" OR AB "lifestyle behaviour*"

OR TI "healthy lifestyle" OR AB "healthy lifestyle"

OR TI "health management" OR AB "health management"

OR TI "self-management" OR AB "self-management"

OR TI "self management" OR AB "self management"

OR TI "self-care" OR AB "self-care"

OR TI "self care" OR AB "self care"

OR TI "medication adherence" OR AB "medication adherence"

OR TI "treatment adherence" OR AB "treatment adherence"

OR TI "health information seeking" OR AB "health information seeking"

OR TI "help-seeking" OR AB "help-seeking"

OR TI "help seeking" OR AB "help seeking"

OR TI "preventive behavior*" OR AB "preventive behavior*"

OR TI "preventive behaviour*" OR AB "preventive behaviour*"

OR TI "vaccine uptake" OR AB "vaccine uptake"

OR TI "screening participation" OR AB "screening participation"

OR TI "physical activity" OR AB "physical activity"

OR TI exercise OR AB exercise

OR TI "physical exercise" OR AB "physical exercise"

)

)

**4. Scopus**

TITLE-ABS-KEY(

"eHealth literacy"

OR "electronic health literacy"

OR "digital health literacy"

OR "online health literacy"

OR eHEALS

OR eHLQ

OR DHLI

)

AND

TITLE-ABS-KEY(

"health behavior*"

OR "health behaviour*"

OR "health-promoting behavior*"

OR "health promoting behavior*"

OR "health promotion behavior*"

OR "health-related behavior*"

OR "health related behavior*"

OR "lifestyle behavior*"

OR "lifestyle behaviour*"

OR "healthy lifestyle"

OR "self-management"

OR "self management"

OR "self-care"

OR "self care"

OR "medication adherence"

OR "treatment adherence"

OR "health information seeking"

OR "preventive behavior*"

OR "preventive behaviour*"

OR "physical activity"

OR exercise

OR "physical exercise"

)

AND PUBYEAR > 2019

AND PUBYEAR < 2027

1. **Web of Science Core Collection**

TS=(

(

"eHealth literacy"

OR "electronic health literacy"

OR "digital health literacy"

OR "online health literacy"

OR "electronic health information literacy"

OR "internet health literacy"

OR "web-based health literacy"

OR "web based health literacy"

OR "mobile health literacy"

OR "mHealth literacy"

OR "digital health competence"

OR "digital health competencies"

OR eHEALS

OR eHLQ

OR DHLI

)

AND

(

"health behavior*"

OR "health behaviour*"

OR "health-promoting behavior*"

OR "health promoting behavior*"

OR "health promotion behavior*"

OR "health-related behavior*"

OR "health related behavior*"

OR "lifestyle behavior*"

OR "lifestyle behaviour*"

OR "healthy lifestyle"

OR "health management"

OR "self-management"

OR "self management"

OR "self-care"

OR "self care"

OR "medication adherence"

OR "treatment adherence"

OR "health information seeking"

OR "help-seeking"

OR "help seeking"

OR "preventive behavior*"

OR "preventive behaviour*"

OR "vaccine uptake"

OR "screening participation"

OR "physical activity"

OR exercise

OR "physical exercise"

)

)
