# Supplementary material for: The Association Between eHealth Literacy and Health Behaviors During and Since the COVID-19 Pandemic: Systematic Review and Meta-Analysis
Source: J Med Internet Res. 2026 Jul 9;28:e94233. doi: 10.2196/94233 (PMC13348804; doi:10.2196/94233)
Supplement: Multimedia Appendix 3 [file jmir-v28-e94233-s003.docx]

To examine the association between eHealth literacy and health behaviours, a forest plot was constructed using effect sizes ORs extracted from eligible studies. Among the included studies:

**1.Directly reported ORs**
To examine the association between eHealth literacy and health behaviours during and since the COVID-19 pandemic, a forest plot was constructed using OR effect sizes extracted from eligible studies. Among the included studies, 9 studies directly reported ORs and corresponding 95% confidence intervals, including 6 studies reporting categorical ORs derived from grouped comparisons of eHealth literacy levels and 3 studies reporting continuous ORs for each unit increase in eHealth literacy score.

**2. Fisher’s z Transformation for Correlation Coefficients**

For studies reporting correlation coefficients (r) without corresponding confidence intervals, 95% confidence intervals were reconstructed using Fisher’s z transformation. A total of ten studies provided correlation coefficients and sample sizes (r and n) and were processed using this approach. The procedures and formulas applied are detailed below.

1. Transformation from r to Fisher’s z

Z= $\frac{1}{2}ln\left( \frac{1+r}{1-r} \right)$

1. Standard error of Fisher’s z

$${SE}_{z}=\frac{1}{\sqrt{n-3}}$$

where n denotes the sample size.

1. Construction of the 95% confidence interval on the z scale

$$z_{lower}=z-1.96\times{SE}_{Z}$$

$$z_{upper}=z+1.96\times{SE}_{Z}$$

2. Back-transformation from Fisher’s z to r

$$r=\frac{e^{2z}-1}{e^{2z}+1}$$

The lower and upper bounds of the 95% confidence interval for r were obtained by applying the inverse transformation to $z_{lower}$ and $z_{upper}$, respectively.
