# Supplementary material for: The Association Between eHealth Literacy and Health Behaviors During and Since the COVID-19 Pandemic: Systematic Review and Meta-Analysis
Source: J Med Internet Res. 2026 Jul 9;28:e94233. doi: 10.2196/94233 (PMC13348804; doi:10.2196/94233)
Supplement: Multimedia Appendix 4 [file jmir-v28-e94233-s004.docx]

### Among the 148 full-text articles excluded from the primary quantitative synthesis, 90 studies reported quantitative effect estimates on the association between eHealth literacy and health behaviors but were not eligible for direct association synthesis because of heterogeneity in effect measure type, outcome operationalization, or analytic design. These studies were classified as effect estimates not eligible for direct association synthesis and were grouped into four categories based on their core methodological characteristics.

### A. Mechanism‑focused Studies

**Definition**: These studies focused on exploring mediating, moderating, or pathway mechanisms rather than reporting the overall direct association between eHealth literacy and health behaviors.

**Forms included**: Mediation models, structural equation modeling (SEM), path analysis, moderation analysis, indirect effect only.

**Number of studies**: n= 30

### B. Multiple Non‑comparable Behavioral Outcomes

**Definition**: These studies reported associations between eHealth literacy and multiple discrete health behaviors or multi‑dimensional behavioral outcomes without providing a single synthesizable direct effect.

**Forms included**: Separate analyses for multiple behaviors, multi‑dimensional behavioral outcomes, no unified direct effect available for meta‑analysis.

**Number of studies**: n = 6

### C. Descriptive or Non‑synthesizable Effect Formats

**Definition**: These studies presented results in descriptive formats, latent profile/class analyses, or between‑group comparisons, without providing effect parameters that could be directly extracted for quantitative synthesis.

**Forms included**: Descriptive proportions, latent profile/class analysis, between‑group comparisons, no usable odds ratio.

**Number of studies**: n = 8

### D. Quantifiable but Non‑synthesizable Effect Formats

**Definition**: These studies provided quantifiable association estimates (e.g., standardized regression coefficients, correlation coefficients, hazard ratios, odds ratios for non‑binary outcomes) that were incompatible with the primary meta‑analysis (which pooled odds ratios for binary outcomes).

**Forms included**: Standardized beta coefficients (β), correlation coefficients (r), hazard ratios (HR), odds ratios for non‑binary outcomes, multiple linear regression coefficients.

**Number of studies**: n = 46
