# Supplementary material for: The Association Between eHealth Literacy and Health Behaviors During and Since the COVID-19 Pandemic: Systematic Review and Meta-Analysis
Source: J Med Internet Res. 2026 Jul 9;28:e94233. doi: 10.2196/94233 (PMC13348804; doi:10.2196/94233)
Supplement: Multimedia Appendix 5 [file jmir-v28-e94233-s005.docx]

**Table S1.** Characteristics of quantitative studies reporting correlation coefficients (r) for the association between eHealth literacy and health behaviors

| **Author** | **Study period** | **Health Behavior Scale** | **Health Behavior Category** | **Specific Health Behavior** | **Research Type** | **r** | **Lower 95% CI** | **Upper 95% CI** | **Geriatric Status** | **Patient Affected Status** | **N** | **Correlation Type** |
| --- | --- | --- | --- | --- | --- | --- | --- | --- | --- | --- | --- | --- |
| Yu et al (2023) [26] | January – August 2022 | DBRS-P, Diabetes Behavior Rating Scale Parent Version | Health management behavior | Diabetes Management Behaviors | Cross-sectional | 0.226 | 0.064 | 0.396 | No | No | 143 | Pearson |
| Choi et al (2021) [32] | April 25 – May 24, 2021 | Healthy lifestyles tool by Cho and Kim | Health promoting behaviors | Healthy Lifestyle | Cross-sectional | 0.52 | 0.411 | 0.614 | No | No | 138 | Pearson |
| Kwon and Oh (2023) [33] | May 28 – June 30, 2021 | Health Promoting Lifestyle Profile II, HPLP II, 52 items | Health promoting behaviors | Health Promoting Behavior | Cross-sectional | 0.347 | 0.247 | 0.44 | No | No | 301 | Pearson |
| Moradi et al (2025) [31] | November 2024 – February 2025 | Health-Promoting Lifestyle Profile II, HPLP-II，49 items | Health promoting behaviors | Healthy Lifestyle | Cross-sectional | 0.565 | 0.501 | 0.624 | No | No | 334 | Spearman |
| Mousazadeh et al (2025) [29] | March – May 2024 | Health-Promoting Lifestyle Profile (HPLP) | Health promoting behaviors | Healthy Lifestyle | Cross-sectional | 0.43 | 0.332 | 0.517 | No | No | 255 | Pearson |
| Rezakhani Moghaddam et al (2022) [30] | September – December 2021 | COVID-19 protective behaviors, 5 items, self-compiled behavioral items | Health promoting behaviors | Healthy Lifestyle | Cross-sectional | 0.285 | 0.189 | 0.375 | No | No | 380 | Pearson |
| Sun et al (2025) [28] | May to September 2024 | non-medication adherence questionnaire for secondary prevention in CHD | Health management behaviors | Non-pharmacological Adherence | Cross-sectional | 0.397 | 0.327 | 0.46 | Yes | Yes | 594 | Spearman |
| Töyer Şahin and Pehlivan (2026) [34] | October 2024 – June 2025 | DSMQ | Health management behaviors | Disease Management | Cross-sectional | 0.505 | 0.397 | 0.599 | No | Yes | 212 | Pearson |
| Li et al (2021) [25] | May 10 – May 20, 2020 | COVID-19–related health behavior questionnaire，15 items | Health promoting behaviors | Healthy Lifestyle | Cross-sectional | 0.476 | 0.44 | 0.509 | No | No | 1873 | Pearson |
| Qian et al (2024) [27] | August 2020 – February 2021 | Gout Patient Self-management Assessment Scale | Health management behavior | Self-Management | Cross-sectional | 0.506 | 0.382 | 0.614 | No | Yes | 168 | Pearson |

**Table S2. Characteristics of quantitative studies reporting grouped odds ratios (ORs) for the association between eHealth literacy and health behaviors**

| **Author** | **Study period** | **Health Behavior Scale** | **Health Behavior Category** | **Specific Health Behavior** | **Research Type** | **Risk of Bias** | **Geriatric Status** | **Patient Affected Status** | **OR** | **Lower 95% CI** | **Upper 95% CI** | **N** |
| --- | --- | --- | --- | --- | --- | --- | --- | --- | --- | --- | --- | --- |
| Kalayou and Awol (2022) [40] | Dec 7, 2021 – Jan 25, 2022 | Not reported | Health decision-making behavior | Administration of COVID-19 vaccine | Cross-sectional | 7 | No | No | 2.7 | 1.7 | 4.1 | 574 |
| Guo et al (2021) [36] | April 9–23, 2020 | Not reported | Health-promoting beHaviors | Wear a surgical mask | Cross-sectional | 9 | No | No | 3.84 | 1.63 | 9.05 | 1501 |
| Hakeem et al (2023) [39] | conducted in 2023 | Not reported | Health-promoting behaviors | Brush | Cross-sectional | 7 | No | Yes | 4.17 | 1.61 | 10 | 478 |
| Guo et al (2024) [37] | November–December 2020 | 4-item Morisky medication adherence | Health management behavior | Medication Adherence | Cross-sectional | 9 | Yes | Yes | 1.55 | 1.08 | 2.11 | 4218 |
| Chau et al (2026) [35] | November 2022 to December 2024 | Mixed, IPAQ-SF for physical activity plus self-reported items for smoking, drinking, and diet. | Health-promoting behaviors | Non-Smoking | Cross-sectional | 8 | Yes | No | 1.67 | 1.05 | 2.63 | 6704 |
| Lee et al (2023) [38] | April, 2020 | WHO and South Korea CDC-revised 10 items of COVID-19 preventive behaviors, with a total score ranging from 0 to 10, unvalidated | Health-promoting behaviors | Preventive Behavior | Cross-sectional | 7 | No | No | 1.99 | 1.51 | 2.62 | 1057 |

**Table S3. Characteristics of quantitative studies reporting continuous correlation coefficients (r) for the association between eHealth literacy and health behaviors**

| **Author** | **Study period** | **Health Behavior Scale** | **Health Behavior Category** | **Specific Health Behavior** | **Research Type** | **Risk of Bias** | **Geriatric Status** | **Patient Affected Status** | **N** | **OR** | **Lower 95% CI** | **Upper 95% CI** |
| --- | --- | --- | --- | --- | --- | --- | --- | --- | --- | --- | --- | --- |
| Jing et al (2021) [41] | August,2020 | self-compiled entry-style measurement | Health-promoting behaviors | Physical Activity | Cross-sectional | 9 | No | No | 4580 | 1.02 | 1.02 | 1.03 |
| Do et al (2020) [42] | April 6–19, 2020 | Classification of self-reported behavioral changes | Health-promoting behaviors | Exercise | Cross-sectional | 9 | No | No | 5209 | 1.04 | 1.03 | 1.05 |
| Tran et al (2022) [43] | April 7 – May 31, 2020 | Single-item self-reported frequency question | Health-promoting behaviors | Hand Hygiene Prevention | Cross-sectional | 8 | No | No | 1851 | 1.18 | 1.15 | 1.22 |
