# Supplementary material for: The Association Between eHealth Literacy and Health Behaviors During and Since the COVID-19 Pandemic: Systematic Review and Meta-Analysis
Source: J Med Internet Res. 2026 Jul 9;28:e94233. doi: 10.2196/94233 (PMC13348804; doi:10.2196/94233)
Supplement: Multimedia Appendix 6 [file jmir-v28-e94233-s006.docx]

**Table S1. Modified NOS risk of bias assessment for studies included in the correlation-based quantitative synthesis**

| **Study** | **Representativeness (1)** | **Sample size justification (1)** | **Nonresponse (1)** | **Exposure assessment (2)** | **Confounding control (2)** | **Outcome assessment (2)** | **Statistical test (1)** | **Total** | **Quality** |
| --- | --- | --- | --- | --- | --- | --- | --- | --- | --- |
| Choi et al (2021) [32] | 0 | 1 | 1 | 2 | 0 | 2 | 1 | 7 | Moderate |
| Qian et al (2024) [27] | 0 | 1 | 1 | 2 | 2 | 2 | 1 | 9 | High |
| Mousazadeh et al (2025) [29] | 1 | 0 | 0 | 2 | 2 | 2 | 1 | 8 | High |
| Kwon and Oh (2023) [33] | 0 | 1 | 1 | 2 | 2 | 2 | 1 | 9 | High |
| Rezakhani Moghaddam et al (2022) [30] | 1 | 0 | 0 | 2 | 2 | 1 | 1 | 7 | Moderate |
| Li et al (2021) [25] | 0 | 0 | 0 | 2 | 2 | 2 | 1 | 7 | Moderate |
| Sun et al (2025) [28] | 1 | 1 | 1 | 2 | 2 | 2 | 1 | 10 | High |
| Moradi et al (2025) [31] | 1 | 1 | 0 | 2 | 2 | 2 | 1 | 9 | High |
| Yu et al (2023) [26] | 0 | 0 | 0 | 2 | 0 | 2 | 1 | 5 | Moderate |
| Töyer Şahin and Pehlivan (2026) [34] | 0 | 1 | 0 | 2 | 2 | 2 | 1 | 8 | High |

**Table S2. Modified NOS risk of bias assessment for studies included in the odds ratio–based quantitative syntheses**

| **Study** | **Representativeness (1)** | **Sample size justification (1)** | **Nonresponse (1)** | **Exposure assessment (2)** | **Confounding control (2)** | **Outcome assessment (2)** | **Statistical test (1)** | **Total (10)** | **Quality** |
| --- | --- | --- | --- | --- | --- | --- | --- | --- | --- |
| Chau et al (2026) [35] | 1 | 0 | 0 | 2 | 2 | 2 | 1 | 8 | High |
| Do et al (2020) [42] | 1 | 0 | 1 | 2 | 2 | 2 | 1 | 9 | High |
| Jing et al (2021) [41] | 1 | 0 | 1 | 2 | 2 | 2 | 1 | 9 | High |
| Guo et al (2024) [37] | 1 | 0 | 1 | 2 | 2 | 2 | 1 | 9 | High |
| Kalayou and Awol (2022) [40] | 1 | 1 | 0 | 1 | 2 | 1 | 1 | 7 | Moderate |
| Guo et al (2021) [36] | 1 | 0 | 1 | 2 | 2 | 2 | 1 | 9 | High |
| Tran et al (2022) [43] | 1 | 0 | 0 | 2 | 2 | 2 | 1 | 8 | High |
| Hakeem et al (2023) [39] | 0 | 0 | 0 | 2 | 2 | 2 | 1 | 7 | Moderate |
| Lee et al (2023) [38] | 0 | 1 | 0 | 2 | 2 | 1 | 1 | 7 | Moderate |
|  |  |  |  |  |  |  |  |  |  |
