# Supplementary material for: The Association Between eHealth Literacy and Health Behaviors During and Since the COVID-19 Pandemic: Systematic Review and Meta-Analysis
Source: J Med Internet Res. 2026 Jul 9;28:e94233. doi: 10.2196/94233 (PMC13348804; doi:10.2196/94233)
Supplement: Multimedia Appendix 7 [file jmir-v28-e94233-s007.docx]

| **Outcome / synthesis** | **Studies, n** | **Participants** | **Effect estimate** | **Heterogeneity / prediction interval** | **Risk of bias** | **Inconsistency** | **Indirectness** | **Imprecision** | **Publication bias** | **Overall certainty** | **Interpretation** |
| --- | --- | --- | --- | --- | --- | --- | --- | --- | --- | --- | --- |
| Correlation-based synthesis | 10 | Not pooled across studies | Pooled r = 0.43, 95% CI 0.36–0.51 | I² = 80.50%; 95% PI 0.22–0.61 | Serious | Serious | Not serious | Not serious | Not strongly suspected, but cannot be ruled out | Very low | Higher eHealth literacy may be associated with more favorable health behaviors, but confidence is very limited because of observational design, risk-of-bias concerns, and substantial heterogeneity. |
| Grouped OR-based synthesis | 6 | Not pooled across studies | Pooled OR = 2.12, 95% CI 1.47–3.05 | I² = 46.33%; 95% PI 1.11–4.06 | Not serious enough to downgrade | Not serious | Not serious | Not serious | Not strongly suspected, but cannot be ruled out | Low | Higher eHealth literacy is probably associated with higher odds of favorable health behaviors, but the evidence remains observational and non-causal. |
| Continuous OR-based synthesis | 3 | Not pooled across studies | Pooled OR = 1.07, 95% CI 0.89–1.30 | I² = 97.98%; 95% PI 0.84–1.37 | Serious | Very serious | Not serious | Serious | Unable to assess reliably | Very low | Evidence for continuous exposure modeling is very uncertain; the pooled estimate was inconclusive and heterogeneity was very high. |

Abbreviations: CI = confidence interval; GRADE = Grading of Recommendations Assessment, Development and Evaluation; OR = odds ratio; PI = prediction interval. The certainty rating refers to confidence in the estimated association between eHealth literacy and health behavior, not to confidence in a causal effect. All included studies were observational and predominantly cross-sectional; therefore, the initial certainty was low. Downgrading was considered across the domains of risk of bias, inconsistency, indirectness, imprecision, and publication bias. No upgrading was applied because the evidence did not meet GRADE criteria for a convincing large effect, clear dose-response relationship, or plausible residual confounding that would reduce the observed association.
