# Supplementary material for: The Association Between eHealth Literacy and Health Behaviors During and Since the COVID-19 Pandemic: Systematic Review and Meta-Analysis
Source: J Med Internet Res. 2026 Jul 9;28:e94233. doi: 10.2196/94233 (PMC13348804; doi:10.2196/94233)
Supplement: Multimedia Appendix 8 [file jmir-v28-e94233-s008.docx]

Funnel plots are presented in **Figures S1-S3.** Visual inspection of **Figure S1** suggested slight asymmetry, but Egger regression did not indicate statistically significant small-study effects for the correlation-based synthesis (**P = .459**). **Figure S2,** corresponding to the grouped OR synthesis, showed some visual asymmetry, but the exploratory Egger regression was not statistically significant (**P = .113**), and the result should be interpreted cautiously given the limited number of studies. **Figure S3,** corresponding to the continuous OR synthesis, was not considered informative because only 3 studies were available.


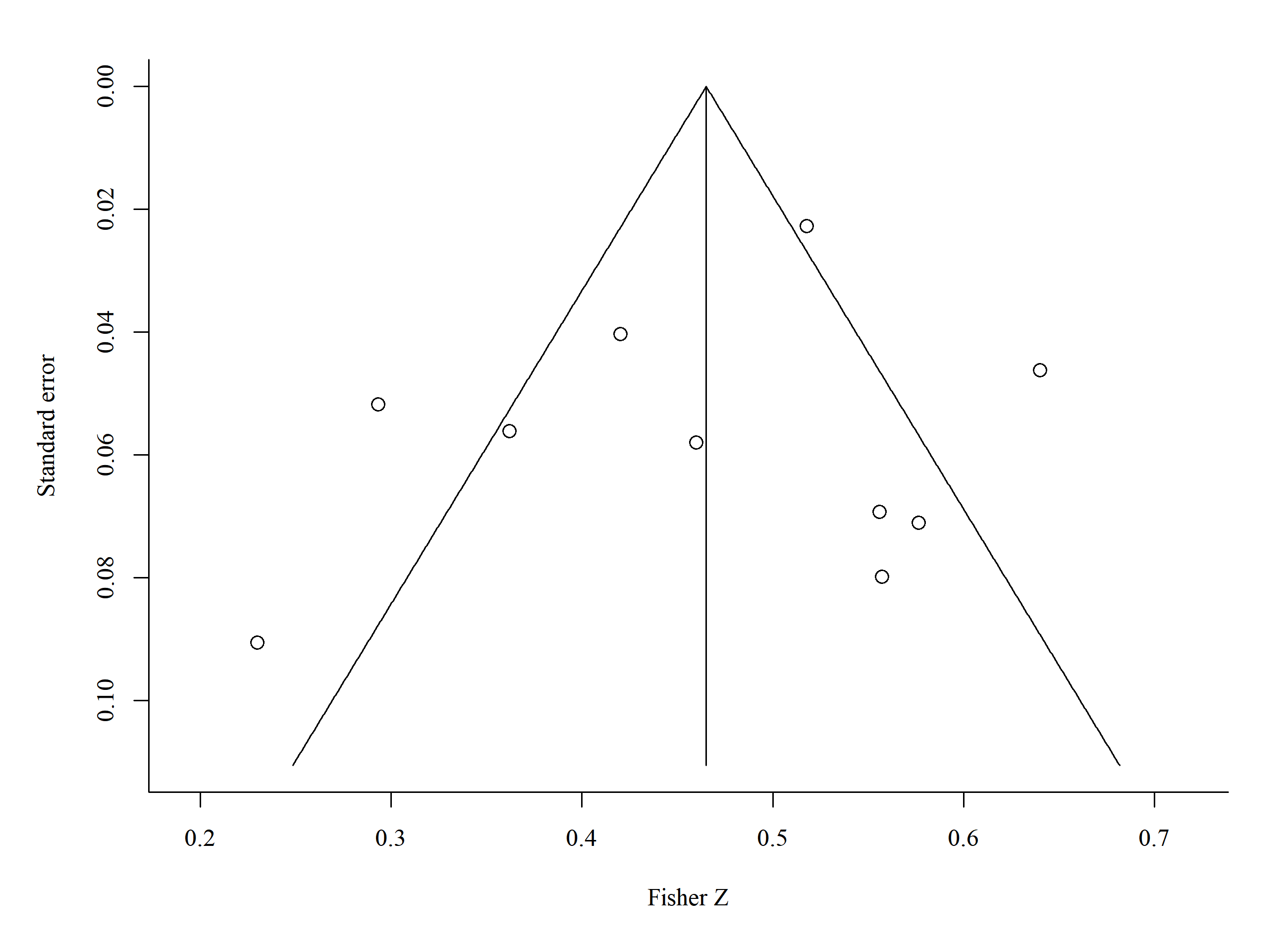


**Figure S1. Funnel plot of correlation coefficients for the association between eHealth literacy and health behaviors.** The horizontal axis shows study-specific effect estimates, and the vertical axis shows their standard errors.


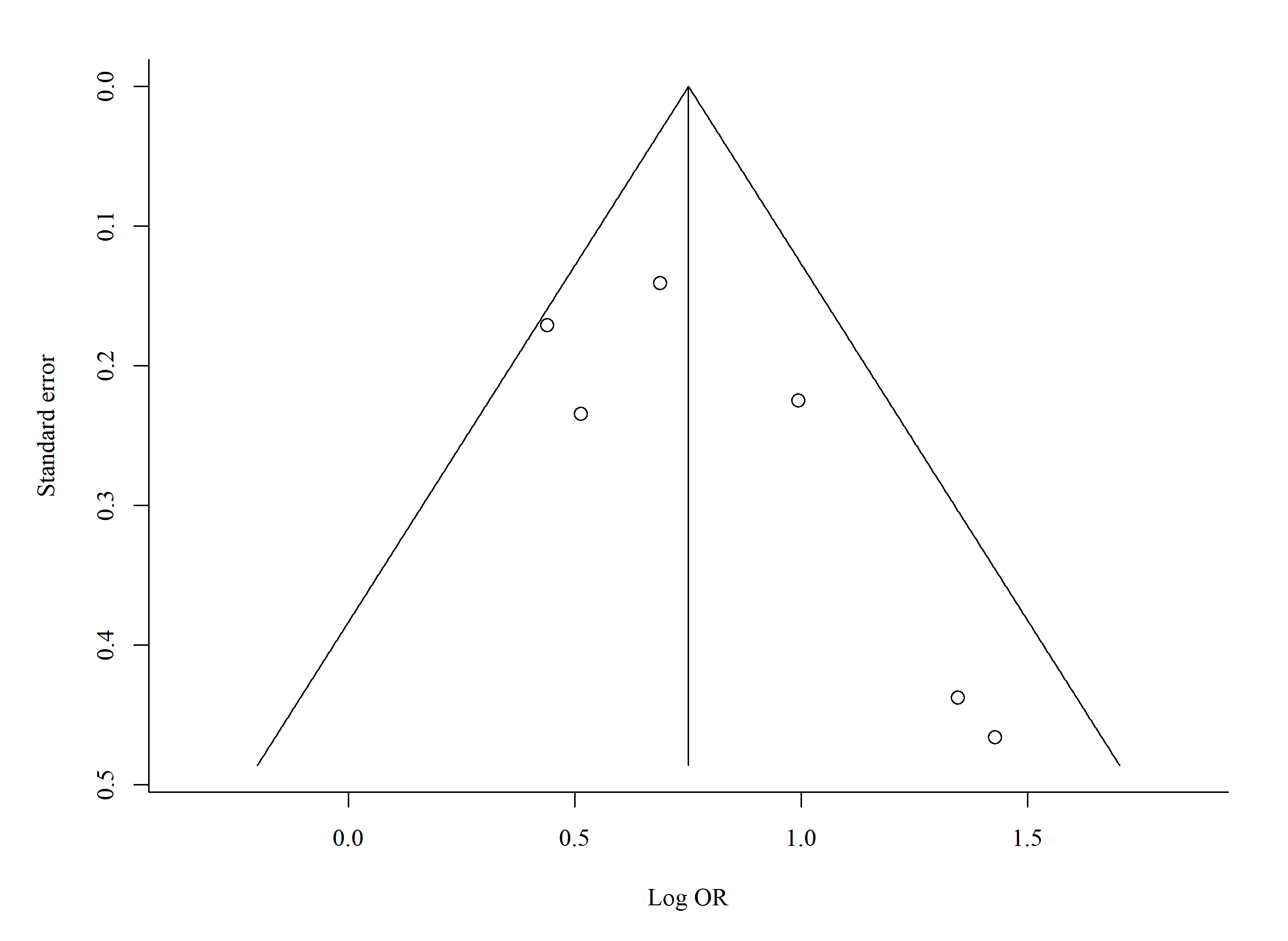


**Figure S2. Funnel plot of grouped odds ratios for the association between eHealth literacy and health behaviors.** The horizontal axis shows study-specific effect estimates, and the vertical axis shows their standard errors.


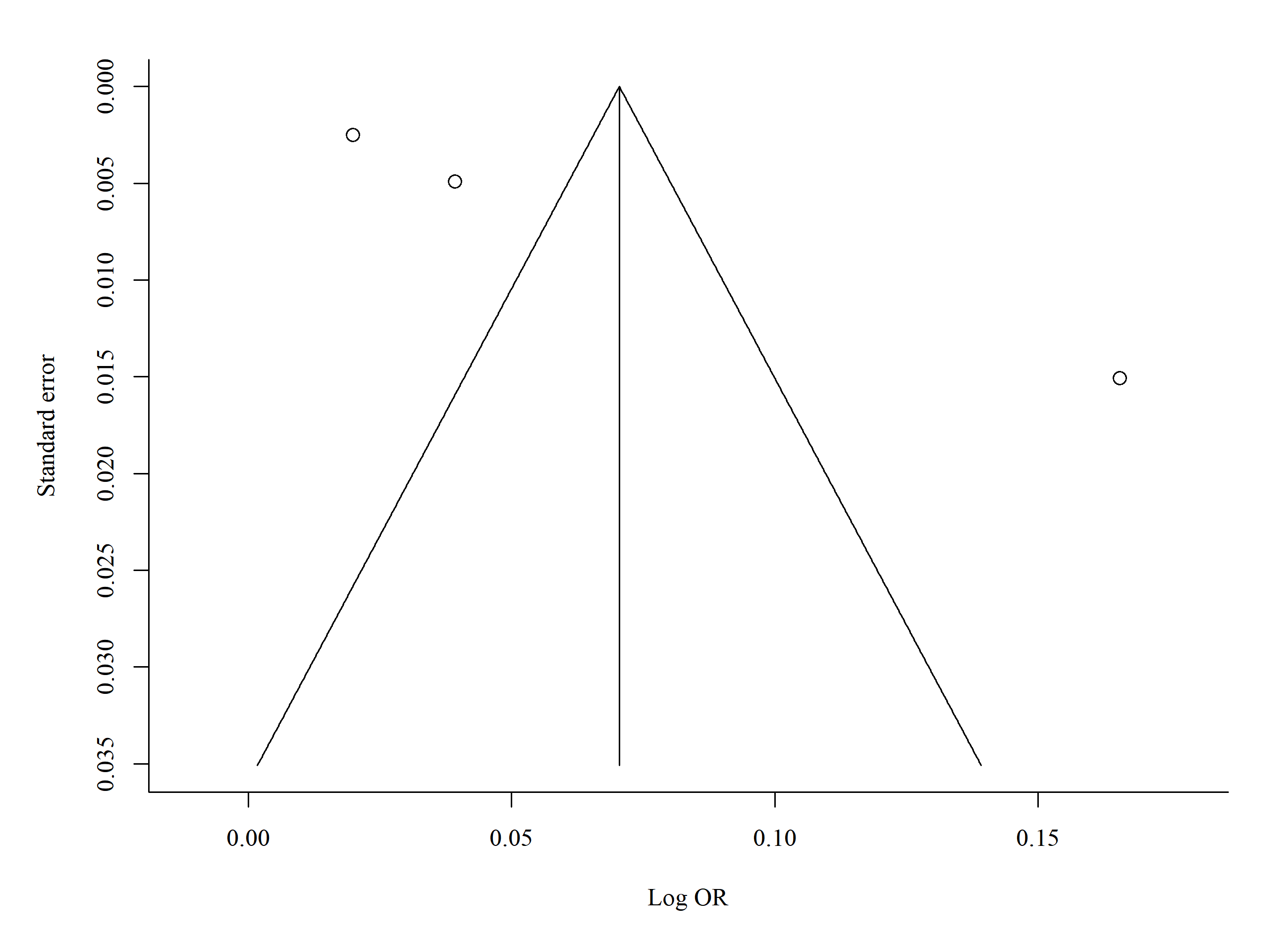


**Figure S3. Funnel plot of continuous odds ratios for the association between eHealth literacy and health behaviors.** The horizontal axis shows study-specific effect estimates, and the vertical axis shows their standard errors.
