# Supplementary material for: The Association Between eHealth Literacy and Health Behaviors During and Since the COVID-19 Pandemic: Systematic Review and Meta-Analysis
Source: J Med Internet Res. 2026 Jul 9;28:e94233. doi: 10.2196/94233 (PMC13348804; doi:10.2196/94233)
Supplement: Multimedia Appendix 9 [file jmir-v28-e94233-s009.docx]

**Table S1. Statistical parameters for subgroup analyses in the correlation-based synthesis**

| **Characteristic** | **Subgroup** | **Studies, k** | **Pooled r** | **CI low** | **CI high** | **P value** | **I² (%)** | **Tau²** | **Q** | **Q p** | **QM** | **QM p** |
| --- | --- | --- | --- | --- | --- | --- | --- | --- | --- | --- | --- | --- |
| Overall | All studies | 10 | 0.43 | 0.37 | 0.49 | <0.001 | 80.5 | 0.01 | 46.16 | <0.001 | — | — |
| Income level | High-income economies | 4 | 0.46 | 0.39 | 0.53 | <0.001 | 63.6 | 0 | 8.24 | 0.04 | 2.97 | 0.09 |
|  | Low- and middle-income economies | 6 | 0.41 | 0.31 | 0.51 | <0.001 | 85.7 | 0.02 | 34.94 | <0.001 |  |  |
| Patient status | Patient populations | 3 | 0.46 | 0.37 | 0.54 | <0.001 | 53.2 | 0 | 4.27 | 0.12 | 0.19 | 0.67 |
|  | General populations | 7 | 0.42 | 0.34 | 0.5 | <0.001 | 85.6 | 0.01 | 41.7 | <0.001 |  |  |
| Geriatric status | Older-adult-focused populations | 1 | 0.4 | 0.33 | 0.46 | <0.001 | NA | NA | NA | NA | 2.82 | 0.09 |
|  | Younger or mixed-age populations | 9 | 0.44 | 0.37 | 0.5 | <0.001 | 81.5 | 0.01 | 43.34 | <0.001 |  |  |
| Health behavior category | Health promoting behaviors | 6 | 0.44 | 0.36 | 0.52 | <0.001 | 85 | 0.01 | 33.4 | <0.001 | 2.19 | 0.14 |
|  | Health management behaviors | 4 | 0.42 | 0.31 | 0.52 | <0.001 | 71.6 | 0.01 | 10.57 | 0.01 |  |  |

Note: r, correlation coefficient; CI, confidence interval; Q, Cochran’s Q statistic for within-subgroup heterogeneity; QM, test statistic for between-subgroup differences; NA, not applicable. All pooled estimates were obtained using a random-effects model. k denotes the number of studies included in each analysis. Heterogeneity statistics were not estimated for subgroups containing only 1 study.

**Table S2. Statistical parameters for subgroup analyses in the grouped OR-based synthesis**

| **Characteristic** | **Subgroup** | **Studies, k** | **Pooled OR** | **CI low** | **CI high** | **P value** | **I² (%)** | **Tau²** | **Q** | **Q p** | **QM** | **QM p** |
| --- | --- | --- | --- | --- | --- | --- | --- | --- | --- | --- | --- | --- |
| Overall | All studies | 6 | 2.12 | 1.64 | 2.74 | <0.001 | 46.33 | 0.04 | 9.32 | 0.1 | — | — |
| Income level | High-income economies | 4 | 2.25 | 1.58 | 3.2 | <0.001 | 41.99 | 0.05 | 5.17 | 0.16 | 0.28 | 0.6 |
|  | Low- and middle-income economies | 2 | 2.01 | 1.17 | 3.45 | 0.01 | 74.15 | 0.11 | 3.87 | 0.05 |  |  |
| Patient status | Patient populations | 2 | 2.31 | 0.89 | 5.99 | 0.08 | 74.86 | 0.37 | 3.98 | 0.05 | 1.09 | 0.3 |
|  | General populations | 4 | 2.18 | 1.68 | 2.83 | <0.001 | 29.31 | 0.02 | 4.24 | 0.24 |  |  |
| Geriatric status | Older-adult-focused populations | 2 | 1.59 | 1.21 | 2.08 | <0.001 | 0 | 0 | 0.07 | 0.8 | 4.7 | 0.03 |
|  | Younger or mixed-age populations | 4 | 2.55 | 1.85 | 3.51 | <0.001 | 34.14 | 0.04 | 4.55 | 0.21 |  |  |
| Health behavior category | Health decision-making behavior | 1 | 2.7 | 1.74 | 4.19 | <0.001 | NA | NA | NA | NA | 4.14 | 0.13 |
|  | Health-promoting behaviors | 4 | 2.25 | 1.58 | 3.2 | <0.001 | 41.99 | 0.05 | 5.17 | 0.16 |  |  |
|  | Health management behavior | 1 | 1.55 | 1.11 | 2.17 | 0.01 | NA | NA | NA | NA |  |  |

Note: OR, odds ratio; CI, confidence interval; Q, Cochran’s Q statistic for within-subgroup heterogeneity; QM, test statistic for between-subgroup differences; NA, not applicable. All pooled estimates were calculated using a random-effects model on the log OR scale and then back-transformed. k indicates the number of studies included in each analysis. Heterogeneity statistics were not estimated for subgroups containing only 1 study.

**Table S3. Statistical parameters for the overall meta-analysis in the continuous OR-based synthesis**

| **Characteristic** | **Subgroup** | **Continuous OR studies, n** | **Pooled OR (95% CI)** | **P value** | **I² (%)** | **Tau²** | **Q** | **Q p** |
| --- | --- | --- | --- | --- | --- | --- | --- | --- |
| Overall | All studies | 3 | 1.07 (1.03-1.12) | 0.001 | 97.98 | 0.00 | 99.00 | <0.001 |

Note: OR, odds ratio; CI, confidence interval. All pooled estimates were calculated using a random-effects model on the log OR scale and then back-transformed. In this table, n indicates the number of studies.
