# Supplementary material for: The Association Between eHealth Literacy and Health Behaviors During and Since the COVID-19 Pandemic: Systematic Review and Meta-Analysis
Source: J Med Internet Res. 2026 Jul 9;28:e94233. doi: 10.2196/94233 (PMC13348804; doi:10.2196/94233)
Supplement: Checklist 1 [file jmir-v28-e94233-s010.docx]

**PRISMA 2020 Checklist**

| **Section and topic** | **Item** | **Checklist item** | **Location in manuscript** |
| --- | --- | --- | --- |
| Title | 1 | Identify the report as a systematic review. | Page 1, lines 1–3 |
| Abstract | 2 | See the PRISMA 2020 for Abstracts checklist. | Page 1, line 8–Page 3, line 11 |
| Introduction | 3 | Describe the rationale for the review in the context of existing knowledge. | Page 4, lines 3–22; Page 5, lines 1–10 |
| Introduction | 4 | Provide an explicit statement of the objective(s) or question(s) the review addresses. | Page 6, lines 1-21 |
| Methods | 5 | Specify the inclusion and exclusion criteria for the review and how studies were grouped for the syntheses. | Page 7, lines 21–22; Page 9, lines 1–3 |
| Methods | 6 | Specify all databases, registers, websites, organisations, reference lists and other sources searched or consulted to identify studies. Specify the date when each source was last searched or consulted. | Page 8, lines 5–14; Multimedia Appendix 2 |
| Methods | 7 | Present the full search strategies for all databases, registers and websites, including any filters and limits used. | Multimedia Appendix 2 |
| Methods | 8 | Specify the methods used to decide whether a study met the inclusion criteria of the review, including screening and selection procedures. | Page 9, lines 11–22 |
| Methods | 9 | Specify the methods used to collect data from reports, including number of reviewers and data extraction processes. | Page 12, lines 6–17 |
| Methods | 10a | List and define all outcomes for which data were sought. Specify methods used to decide which results to collect. | Page 10, lines 1–22; Table 1 |
| Methods | 10b | List and define all other variables for which data were sought. Describe assumptions about missing or unclear information. | Page 10, lines 19–22; Page 11, lines 1–4 |
| Methods | 11 | Specify the methods used to assess risk of bias in the included studies, including details of the tool(s) used and reviewer procedures. | Page 11, lines 7–11; Page 12, lines 1–3 |
| Methods | 12 | Specify for each outcome the effect measure(s) used in the synthesis. | Page 12, lines 19–22; Page 13, lines 1–20 |
| Methods | 13a | Describe the processes used to decide which studies were eligible for each synthesis. | Page 8, lines 8–17; |
| Methods | 13b | Describe any methods required to prepare the data for presentation or synthesis. | Page 12, lines 19–22; Page 13, lines 1–20; Multimedia Appendix 3 |
| Methods | 13c | Describe any methods used to tabulate or visually display results of individual studies and syntheses. | Page 14, lines 1–4; Figures 2–5; Multimedia Appendices 5–8 |
| Methods | 13d | Describe any methods used to synthesise results and provide rationale. | Page 14, lines 5–6 |
| Methods | 13e | Describe any methods used to explore possible causes of heterogeneity among study results. | Page 14, lines 8–13; Page 15, lines 16–19 |
| Methods | 13f | Describe any sensitivity analyses conducted to assess robustness of the synthesised results. | Page 14, lines 14–17; |
| Methods | 14 | Describe any methods used to assess risk of bias due to missing results in a synthesis. | Page 16, lines 10–12; |
| Methods | 15 | Describe any methods used to assess certainty in the body of evidence. | Page 16; Page 17, lines 1–3 |
| Results | 16a | Describe the results of the search and selection process, ideally using a flow diagram. | Page 17, lines 18–22; Page 18 |
| Results | 16b | Cite studies that might appear to meet the inclusion criteria, but which were excluded, and explain why they were excluded. | Page 18, lines 1–15; Figure 1; Multimedia Appendix 4 |
| Results | 17 | Cite each included study and present its characteristics. | Page 20–23; Table 2; Multimedia Appendix 5 |
| Results | 18 | Present assessments of risk of bias for each included study. | Page 26, lines 1–11; Figure 2; Multimedia Appendix 6 |
| Results | 19 | For all outcomes, present, for each study: summary statistics and an effect estimate with precision. | Page 28–32; Figures 3–5; Multimedia Appendix 8 |
| Results | 20a | For each synthesis, briefly summarise the characteristics and risk of bias among contributing studies. | Table 2; Figure 2; Multimedia Appendices 5–6 |
| Results | 20b | Present results of all statistical syntheses conducted, including summary estimates, precision, and measures of heterogeneity. | Page 28–32; Figures 3–5 |
| Results | 20c | Present results of all investigations of possible causes of heterogeneity among study results. | Page 33–37; Table 3; Multimedia Appendix 8 |
| Results | 20d | Present results of all sensitivity analyses conducted to assess robustness of the synthesised results. | Page 32, lines 10–14; Page 33, lines 1–16; Multimedia Appendix 7 |
| Results | 21 | Present assessments of risk of bias due to missing results for each synthesis. | Page 33, lines 12–15; Multimedia Appendix 7 |
| Results | 22 | Present assessments of certainty in the body of evidence for each outcome. | Page 26-27;Table S2 |
| Discussion | 23a | Provide a general interpretation of the results in the context of other evidence. | Page 38, lines 1–13 |
| Discussion | 23b | Discuss any limitations of the evidence included in the review. | Page 40, lines 11–22; Page 41, lines 1–9; |
| Discussion | 23c | Discuss any limitations of the review processes used. | Page 41, lines 10–17 |
| Discussion | 23d | Discuss implications of the results for practice, policy, and future research. | Page 41, lines 19–22; Page 47 |
| Other information | 24a | Provide registration information for the review. | Page 7, lines 1–3 |
| Other information | 24b | Indicate where the review protocol can be accessed, or state that a protocol was not prepared. | Page 7, lines 1-3 |
| Other information | 24c | Describe and explain any amendments to information provided at registration or in the protocol. | Page 17, lines 4–15 |
| Other information | 25 | Describe sources of financial or non-financial support for the review. | Abstract: Page 3, lines 7–9; Other Information: Page 53, lines 5–7 |
| Other information | 26 | Declare any competing interests of review authors. | Page 52, lines 18–19 |
| Other information | 27 | Report which materials are publicly available and where they can be found. | Page 53, lines 1–3 |
