# Supplementary material for: The Association Between eHealth Literacy and Health Behaviors During and Since the COVID-19 Pandemic: Systematic Review and Meta-Analysis
Source: J Med Internet Res. 2026 Jul 9;28:e94233. doi: 10.2196/94233 (PMC13348804; doi:10.2196/94233)
Supplement: Checklist 2 [file jmir-v28-e94233-s011.docx]

**PRISMA-S Extension Checklist**

| **Section/topic** | **Item** | **Checklist item** | **Location / response for this review** |
| --- | --- | --- | --- |
| Information sources and methods | 1. Database name | Name each individual database searched, stating the platform for each. | Methods; Multimedia Appendix 2. PubMed (MEDLINE/NLM), Embase (Elsevier), Web of Science Core Collection (Clarivate), CINAHL Ultimate (EBSCOhost), and Scopus (Elsevier). |
| Information sources and methods | 2. Multi-database searching | If databases were searched simultaneously on a single platform, state the name of the platform and databases. | Not applicable; databases were searched separately. |
| Information sources and methods | 3. Study registries | List any study registries searched. | No study registries were systematically searched because the review focused on observational studies reporting extractable association estimates. |
| Information sources and methods | 4. Online resources and browsing | Describe online/print sources purposefully searched or browsed and how this was done. | Grey literature, preprints, dissertations, conference proceedings, and organizational websites were not systematically searched. Reference lists of included studies and relevant reviews were manually screened. |
| Information sources and methods | 5. Citation searching | Indicate whether cited or citing references were examined and describe methods. | Reference-list screening of included studies and relevant reviews and forward citation tracking were conducted up to March 27, 2026. No additional eligible records were identified. |
| Information sources and methods | 6. Contacts | Indicate whether additional studies/data were sought by contacting authors or others. | Authors and experts were not contacted to identify additional studies. Reports not retrieved were recorded. |
| Information sources and methods | 7. Other methods | Describe any additional information sources or search methods used. | No additional search methods were used beyond database searching, reference-list screening, and forward citation tracking. |
| Search strategies | 8. Full search strategies | Include the search strategies for each database and information source, copied and pasted exactly as run. | Full line-by-line search strategies for all databases are provided in Multimedia Appendix 2. |
| Search strategies | 9. Limits and restrictions | Specify no limits or describe limits/restrictions and justify. | No language restrictions were applied at the search stage. Retrieval was limited to publications from 2020 onward for feasibility, and final eligibility was based on data-collection timing during or since the COVID-19 period. Database-specific limits are reported in Multimedia Appendix 2. |
| Search strategies | 10. Search filters | Indicate whether published search filters were used and cite them. | No published methodological search filter was used. |
| Search strategies | 11. Prior work | Indicate when strategies from prior reviews were adapted/reused and cite. | Search terms were developed for this review. Prior reviews informed conceptual orientation, but no complete search strategy from a prior review was reused. |
| Search strategies | 12. Updates | Report methods used to update searches. | Searches were last run or updated on March 27, 2026. No automated email-alert update was used after the final search. |
| Search strategies | 13. Dates of searches | For each strategy, provide the date when the last search occurred. | The last search date was March 27, 2026 for all databases; dates are repeated under each database strategy in Multimedia Appendix 2. |
| Peer review | 14. Peer review | Describe any search peer review process. | The search strategy was developed by the review team and was not externally peer reviewed using the PRESS checklist. |
| Managing records | 15. Total records | Document total records identified from each database and other source. | Database search: PubMed 542, Embase 565, Web of Science Core Collection 478, CINAHL Ultimate 155, and Scopus 682; total database records 2422. Other methods: reference-list screening 0 and forward citation tracking 0 additional records. See Figure 1. |
| Managing records | 16. Deduplication | Describe processes and any software used to deduplicate records. | Records were imported into EndNote X9. Duplicates were removed using the EndNote duplicate-detection function and checked manually based on title, authors, year, journal, DOI, and abstract. Duplicate records removed: n=1153. |
